# The signal peptide plus a cluster of positive charges in prion protein dictate chaperone-mediated Sec61 channel gating

**DOI:** 10.1242/bio.040691

**Published:** 2019-02-11

**Authors:** Anke Ziska, Jörg Tatzelt, Johanna Dudek, Adrienne W. Paton, James C. Paton, Richard Zimmermann, Sarah Haßdenteufel

**Affiliations:** 1 Department of Medical Biochemistry and Molecular Biology, Saarland University, 66421 Homburg, Germany; 2 Department Biochemistry of Neurodegenerative Diseases, Institute of Biochemistry and Pathobiochemistry, Ruhr University, 44801 Bochum, Germany; 3 School of Molecular and Biomedical Sciences, Research Centre for Infectious Disease, University of Adelaide, Adelaide, South Australia 5005, Australia

**Keywords:** Protein targeting and translocation, BiP, Sec63, Cluster of positive charges, Mature region, Signal peptide

## Abstract

The Sec61-complex as a dynamic polypeptide-conducting channel mediates protein transport into the human endoplasmic reticulum (ER) with the help of additional components. ER membrane resident Hsp40-type co-chaperone Sec63 as well as the ER lumenal Hsp70-type chaperone BiP were proposed to facilitate channel opening in a precursor-specific fashion. Here, we report on their rules of engagement in ER import of the prion protein (PrP) by addressing sixteen PrP-related variants which differ in their signal peptides and mature parts, respectively. Transport into the ER of semi-permeabilized human cells was analyzed upon depletion of the components by siRNA- or toxin-treatment. The results are consistent with the view of separate functions of BiP and Sec63 and strongly suggest that the co-chaperone/chaperone-pair facilitates Sec61 channel gating to the open state when precursor polypeptides with weak signal peptides in combination with detrimental features in the adjacent mature part were targeted. Thus, we expand the view of chaperone-mediated Sec61 channel gating by providing a novel example of a polybasic motif that interferes with signal peptide-mediated Sec61 channel gating.

This article has an associated First Person interview with the first author of the paper.

## Abstract (precis)

**Summary:** We present a hitherto overlooked polybasic motif as functional domain in the early mature region of prion protein which interferes with ER import and requires compensation by chaperone-mediated Sec61 gating.

## INTRODUCTION

The endoplasmic reticulum (ER) represents the major site of membrane and secretory protein biogenesis of the mammalian cell. Such cellular organization and functional specialization require mechanisms for both, directed delivery of precursor polypeptides to their destination in the cell, and then regulated transport across the membrane barrier. Failed ER import and subsequent cytosolic aggregation or integration into the ER membrane with an unusual topology is, in the case of the prion protein (PrP), associated with neurotoxicity (Hegde et al., 1998a; Ma et al., 2002; Miesbauer et al., 2010; Rambold et al., 2006; Tatzelt and Winklhofer, 2004). However, a hallmark of prion diseases, such as Creutzfeldt Jakob disease, is the conversion of the cellular prion protein PrP^C^ into the misfolded isoform PrP^Sc^. As a main component of infectious prions, it is responsible for prion propagation (Collinge, 2001; Prusiner et al., 1998). Thus, the normally GPI-anchored plasma membrane protein shows an unusual complex topology with a soluble and two membrane integrated forms driven by various signals in the PrP sequence.

Delivery to the ER involves the interplay between signals of the precursor polypeptide and corresponding targeting factors within the cytosol and ER membrane. Cleavable N-terminal signal peptides (SP) ideally function as identity tags without affecting the encoded information of the mature protein. SPs typically consist of 20–30 amino acid residues with a three-domain structure. The positively charged amino terminal (N-)region is followed by a central hydrophobic (H-)region and a slightly polar carboxy terminal (C-)region, which includes the recognition site for the signal peptidase (Hegde and Bernstein, 2006; von Heijne, 1985). Thus, entry into the ER lumen can be monitored by cleavage of the N-terminal SP and/or N-glycosylation of respective sites within the mature region or a fusion tag (OPG). Furthermore, targeting is driven by internal transmembrane domains (TMDs) or the C-terminal anchor sequence of Glycosylphosphatidylinositol-(GPI)-anchored proteins (Ast et al., 2013). Internal signals additionally encode topological information. The decision of whether the C- or N-terminus is translocated is typically made in response to charges present in the signal and the translocation channel according to the positive-inside rule (Goder et al., 2004; Junne et al., 2007).

However, hydrophobicity is a central feature and important driver for recognition of all ER signal peptides irrespective of their origin or nature. Classically, the SP is bound by the signal recognition particle (SRP) and, with the help of the heterodimeric ER membrane resident SRP receptor (SR), the arrested precursor polypeptide is targeted to the Sec61 polypeptide-conducting channel. Depletion of the catalytic alpha-subunit of SR interferes with transfer of the ribosome-bound nascent chain to the pore and causes shutdown of the SRP-SR targeting route. Recent findings expanded the view of protein targeting to the ER by the additional ER membrane receptors Sec62 and hSnd2, both originally identified in yeast. Sec62 represents the general route for targeting of yeast GPI-anchored proteins; however, so far PrP is the only human representative found to involve Sec62 in a genetic screen (Davis et al., 2015). Snd2 also served substrates with internal signals in yeast and managed an inhomogeneous range of substrates in human cells (Aviram et al., 2016; Haßdenteufel et al., 2017).

Irrespectively of the targeting strategy, all routes into the ER, typically, converge at the Sec61 complex for import of the targeted polypeptides into the ER. The ten transmembrane helices of the pore-forming alpha-subunit of Sec61 are arranged to form two halves moving in a dynamic equilibrium between opened and closed conformations. Flexibility of the channel is required for regulated entry of presecretory proteins into the ER lumen and maintenance of the ion permeability barrier with respect to the storage of calcium within the ER. Current understanding of channel opening, referred to as Sec61 gating, is described as a two-step mechanism. Priming leads to destabilization of the closed conformation by docking of the ribosome to cytosolic loops of Sec61. Subsequent opening and stabilization of the conformation involves the SP. Therefore, a second recognition step takes place, again involving interaction between the SP and residues within the hourglass-shaped pore. Effective insertion of the signal results in the opening of both the lateral gate for release of the SP into the membrane and the luminal gate for passage of the nascent chain through the aqueous pore into the ER lumen. Consequently, transition from the closed to the opened state requires loosening of respective channel interactions (Trueman et al., 2011). Thus, translocation capacity of the SP is, on the one hand, defined by its capacity for intercalation into the lateral gate and replacement of helix 2 (Voorhees and Hegde, 2016) and, on the other hand, it depends on the mode of insertion into the channel. Typically, soluble precursor polypeptides adopt a loop-structure with the tip of the SP oriented towards the cytosol (Shaw et al., 1988).

The PrP-derived SP is supposed to have a weak capacity in terms of channel gating which was characterized to also depend on the context of its authentic mature part (Kim and Hegde, 2002; Pfeiffer et al., 2013). The definition of properties relevant for Sec61 gating was complicated by the multiple layers of information encoded in a single SP (Hegde, 2002). However, weakness of the PrP-SP was reflected by an extended dwell time of the SP at the cytosolic face of the Sec61 channel, weak binding of the ribosome to the translocon and a delay in translocation (Conti et al., 2015; Rane et al., 2010).

ER membrane integral Hsp40-co-chaperone Sec63 and ER luminal Hsp70-chaperone BiP facilitate Sec61 gating in a precursor-specific fashion, also that of PrP (Lang et al., 2012; Schäuble et al., 2012). While a weak SP or detrimental mature region determined engagement of BiP and/or Sec63 in case of small presecretory proteins (Haßdenteufel et al., 2018; Johnson et al., 2013), rules for engagement in ER import of PrP remained elusive. Here, we set out to define what is causing its deficiency. To address a variety of precursor characteristics we made use of the modular structure of PrP. A set of PrP-related precursor polypeptides, each differing in the signal peptide or mature region, was subjected to our established approach of siRNA- or toxin-mediated depletion of the components and *in vitro* protein import into digitonin-permeabilized human cells. The data supported the current view of multiple Sec63 and BiP functions, each dictated by different precursor characteristics. We found a polybasic motif in the early PrP mature region to determine requirement for BiP when combined with a weak SP. We propose that in the presence of signal peptides with basic amino acid residues at the N-terminus and an apolar C-region, the Sec61 channel switches spontaneously towards the open state as in case of preprolactin (ppl). In contrast, signals lacking these characteristics and having adjacent detrimental features in the mature region may rely on accessory components, assisting in channel gating as in case of PrP. Thus, we expand the model of chaperone-mediated Sec61 gating by providing a second, mechanistically different example of a functional mature domain, which interferes with loop-insertion and SP-mediated Sec61 gating.

## RESULTS

### Depletion of BiP inhibits ER import of prion protein due to the signal peptide

**Fig. 1.**
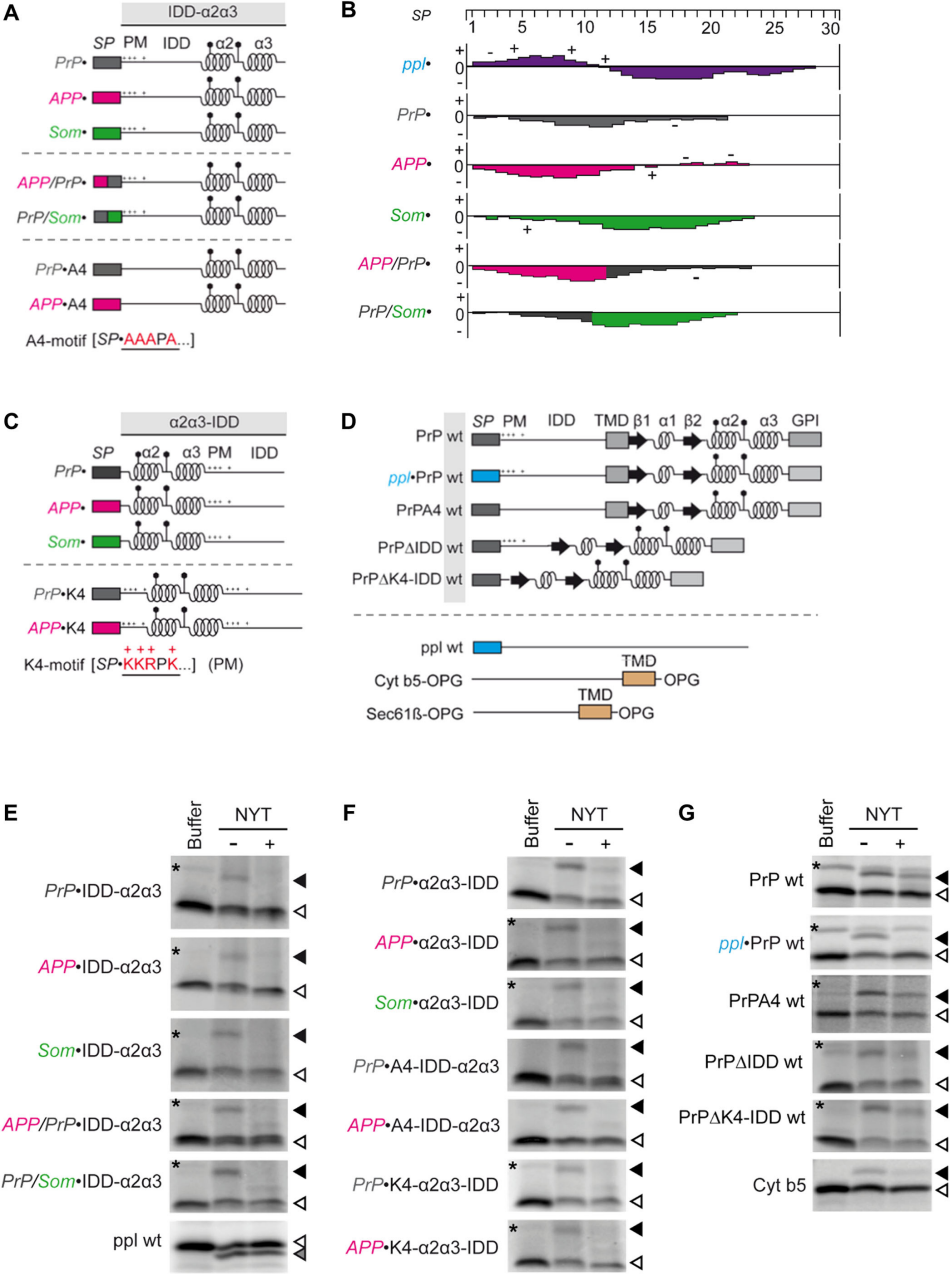
**Model precursor proteins.** (A,C,D) Schematic representation of the constructs used in this study. (A) IDD-α2α3 variants. (C) α2α3-IDD variants. (D) PrP wt variants and control precursor polypeptides. *SP*, signal peptide; PM, polybasic motif (+); IDD, intrinsically disordered domain; α2α3, α-helical regions 2 and 3; β, beta-sheet; lollipops, N-glycans; TMD, transmembrane domain; GPI, glycosylphosphatidylinositol. (B) Kyte-Doolittle Hydrophobicity Plots of the signal peptides used in this study. Charged amino acid residues are indicated (-/+). Scale: −4.5 to +4.5. (E–G) The indicated PrP variants and control precursor polypeptides were synthesized in reticulocyte lysate in the absence (i.e. presence of buffer) or presence of membranes and the tripeptide NYT (-/+), respectively. (E) IDD-α2α3 variants and ppl. (F) Variants of α2α3-IDD, K4-α2α3-IDD and A4-IDD-α2α3. (G) PrP wt variants, Cyt b5-OPG and Sec61β-OPG. All samples (E–G) were subjected to SDS-PAGE and phosphorimaging. Relevant parts of the phosphorimages are shown. Filled triangle, glycosylated protein; unfilled triangle: precursor polypeptide; star, putatively ubiquitinated precursor polypeptide (Rane et al., 2008); PrP, prion protein; APP, amyloid precursor protein; Som, somatostatin; ppl, preprolactin; Cyt b5, Cytochrome b5; OPG, opsin-derived sequence with N-glycosylation site; wt, wild type. See also Tables S1 and S2 and Fig. S1. For complete phosphorimages, see Figs S4 and S5.

The PrP-derived signal peptide is believed to have a weak Sec61 channel gating capacity compared to the SP of ppl (Rutkowski et al., 2001). Therefore, the PrP precursor relies on auxiliary components of the Sec61 translocation machinery, such as the ER luminal Hsp70-chaperone BiP (Lang et al., 2012; Schäuble et al., 2012). In light of recent novel insights into the rules for engagement of BiP in translocation of small presecretory proteins (Johnson et al., 2013), we aimed to evaluate the determinants for BiP assistance in PrP transport. To address this issue, we made use of three different sets of PrP-related precursor polypeptides (Fig. 1A,C,D; Table S1) (Pfeiffer et al., 2013). They vary in the preceding SP as well as the composition of the mature region. All PrP-related precursor proteins, as well as the control model proteins ppl (SRP-dependent and Sec61-dependent) and Cyt b5-OPG (Sec61-independent), were synthesized in the presence of [^35^S]methionine and ER membranes and in the simultaneous presence or absence of the N-glycosylation tripeptide inhibitor NYT. For visualization, samples were subjected to SDS-PAGE and phosphorimaging. Accordingly, the comparison of the bands produced under plus or minus NYT conditions allowed the identification of N-glycosylated proteins (Fig. 1E–G). Modification occurred on either endogenous sites (PrP variants) or a C-terminal opsin-derived tag (OPG-tag of tail-anchored proteins).

**Fig. 2.**
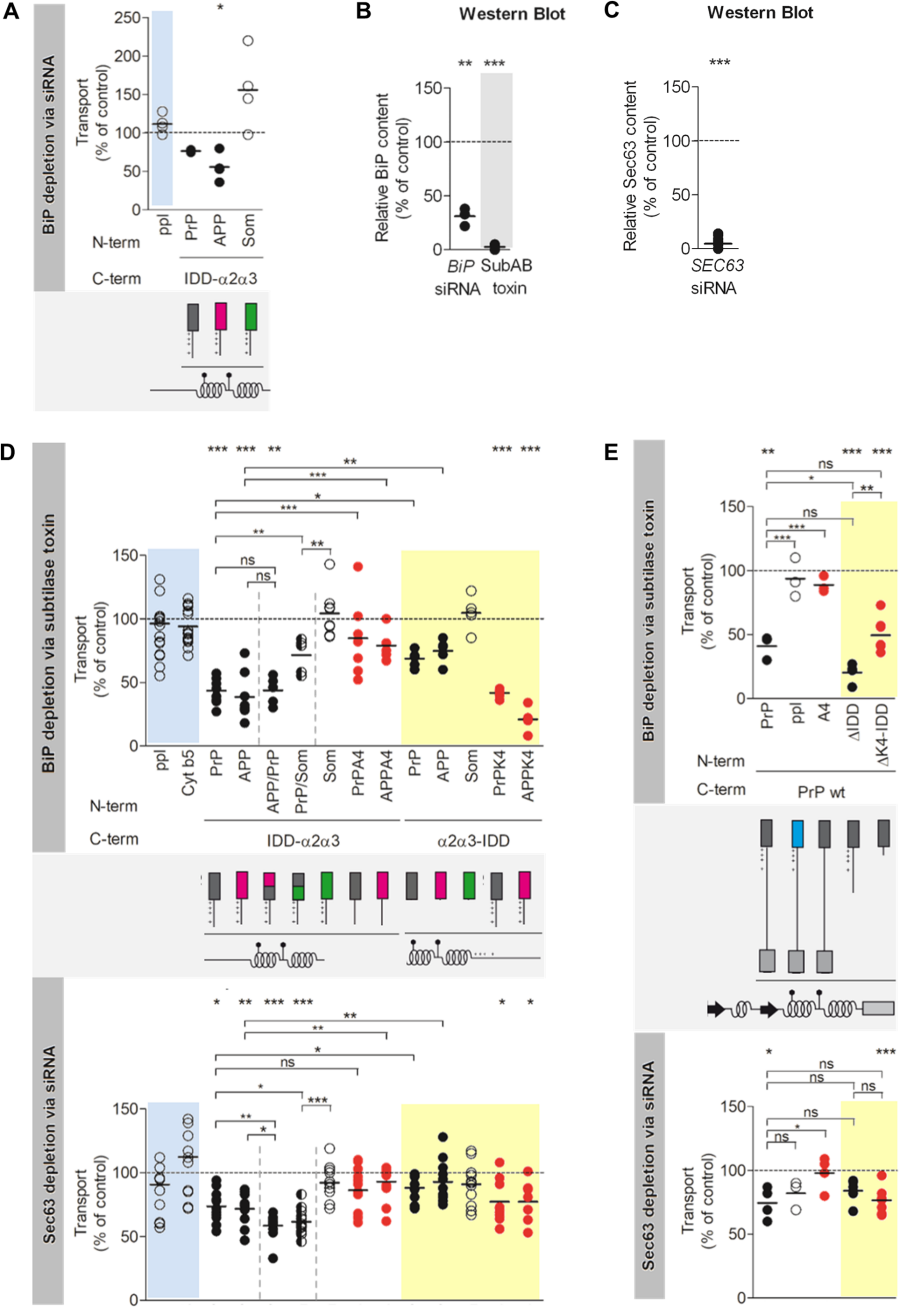
**Engagement of BiP and Sec63 in ER import of prion protein is differentially determined.** For protein depletion, HeLa cells were treated with the corresponding siRNA (Table S3) or subtilase toxin, as indicated. After digitonin-permeabilization of the harvested cells (A–E), reticulocyte lysate was programmed with the indicated precursor polypeptides and incubated in the absence or presence of depleted or control ER membranes (A,D–E). Radioactive samples were subjected to SDS-PAGE and phosphorimaging (Fig. S2A,D–I). Transport efficiencies were calculated as the proportion of N-glycosylation or signal peptide cleavage of the total amount of synthesized precursors with the individual control sample set to 100%. Individual data points and the mean of at least three individual experiments are shown. For statistical analysis (***P<0.001, **P<0.01, *P<0.05), a Student's *t*-test (upper row of stars) or ANOVA with the Dunnett's and Newman–Keuls post hoc test, respectively, were performed (horizontal brackets). (A) *BiP* siRNA effects on transport efficiency of ppl and IDD-α2α3 variants with various SPs. (D,E, upper dot plots) Subtilase toxin effects on transport efficiency of IDD-α2α3 and α2α3-IDD variants (D) as well as PrP wt variants (E). (D,E, bottom dot plots) *SEC63* siRNA effects on transport efficiency of IDD-α2α3 and α2α3-IDD variants (D) as well as PrP wt variants (E). (B,C) Protein content of HeLa cells depleted of BiP (B) or Sec63 (C) relative to β-actin was validated by western blot and the indicated antibodies (Fig. S2B,C). The control sample was set to 100%. Filled dots, weak SPs; unfilled dots, strong SPs and controls; red dots, charge variants; yellow panel, structural variants; blue panel, control precursor polypeptides. For complete phosphorimages, see Figs S6–S10.

Having established ER transport of our model precursor proteins, we investigated the translocation requirements of the first set of PrP variants (Fig. 1A). Each precursor is equipped with a different ER signal peptide either derived of PrP, amyloid precursor protein (APP) or somatostatin (Som) (Fig. 1B). The SP precedes a minimal unit of the PrP mature region, called IDD-α2α3, composed of the intrinsically disordered domain (IDD) and C-terminal alpha-helices (α2α3). Other domains, such as the TMD or GPI anchor sequence, are lacking in favor of topological homogeneity and full import into the ER (Kim and Hegde, 2002; Miesbauer et al., 2009). The respective set of PrP-related SP-chimera (IDD-α2α3) was subjected to an established protocol for *in vitro* protein translocation into the ER of semi-permeabilized HeLa cells upon siRNA-mediated gene silencing of BiP (Table S3) (Haßdenteufel et al., 2018). Cells were treated for 48 h with *BIP*-targeting or control siRNA before digitonin-permeabilization and used as an ER membrane source in rabbit reticulocyte lysate. Precursor polypeptides were synthesized in the presence of [^35^S]methionine and ER membranes. For visualization, samples were subjected to SDS-PAGE and phosphorimaging. Signal peptide cleavage (ppl) or N-glycosylation (PrP variants) reported about translocation efficiency when quantified in comparison to negative control siRNA treated cells. Silencing efficiency was evaluated by western blot with established antibodies (Haßdenteufel et al., 2018). It was previously established that these depletion conditions lead to >70% BiP depletion, without substantially affecting cell growth, cell viability, ER/cell morphology, and ER protein import components (Schäuble et al., 2012; Haßdenteufel et al., 2018). After 48 h treatment with *BiP* siRNA, the protein content of BiP was reduced to 30% compared to control cells as expected (Fig. 2B; Fig. S2B). Although siRNA-mediated BiP depletion was rather incomplete, moderate effects on translocation of IDD-α2α3 were observed (Fig. 2A, white panel; Fig. S2A). However, glycosylation efficiency was selectively inhibited in the presence of the PrP- or APP-SP but in the presence of the Som-SP it was not. In addition, ppl transport was not affected (Fig. 2A, blue panel; Fig. S2A).

Driven by this finding, we changed to an alternative strategy for highly efficient reduction of BiP content by subtilase AB (SubAB) cytotoxin (Paton et al., 2006; Schäuble et al., 2012). Strikingly, 2 h treatment of HeLa cells with SubAB before semi-permeabilization resulted in 97% knockdown of BiP and strengthened translocation defects compared with the siRNA approach (Fig. 2B,D, white panel; Fig. S2B). Transport of the negative control ppl was as efficient in SubAB treated cells as in control cells treated with inactive Sub_A272_B toxin (Fig. 2D, blue panel; Fig. S2D). Insertion efficiency of the model tail-anchored protein Cyt b5-OPG (Sec61-independent) was assayed under posttranslational conditions, i.e. after completion of protein synthesis, demonstrating integrity of the analyzed ER membranes (Fig. 2D, blue panel; Fig. S2F).

Since BiP-dependence of PrP translocation was shown on a PrP-related precursor variant, the precursor of the wild-type (wt) protein was subjected next to the same subtilase approach (Fig. 1D; Table S1). Translocation of PrP wt showed the same perturbation upon BiP cleavage as PrP-IDD-α2α3 (Fig. 2E, white panel; Fig. S2D,F). Here, too, exchange of the signal peptide by that derived of a BiP-independent substrate, such as ppl, led to BiP-independent translocation of the PrP wt mature region (Fig. 2E, white panel; Fig. S2F).

In sum, the presented data argue for signal peptide-specific assistance of protein translocation by BiP. Having the PrP- and APP-SP identified as BiP-dependent and the Som- and ppl-SP as BiP-independent, the question arises: how do they differ and what defines BiP dependence? Consequent computational analysis of our model signal peptides indeed demonstrated differences in the overall hydrophobicity (ΔG^pred^) and charge load as well as the probability for loop-insertion (N-in^pred^) (Table S2). Furthermore, the distribution of basic and apolar amino acid residues along the sequences varied (Fig. 1B; Table S1). Both PrP and APP show accumulation of apolar residues at the N-terminus whereas positively charged amino acids are missing. In addition to the high N- in values of Som- and ppl-, we note that the positive charges at the N-terminus and the highly hydrophobic middle part may define the two SPs as ‘strong’ in terms of channel gating. To experimentally address this point, we took advantage of two chimeric signal peptides composed of the N-terminal half of either APP or PrP and the C-terminal half of either PrP (APP/PrP) or Som (PrP/Som) (Fig. 1A,B; Tables S1, S2) (Pfeiffer et al., 2013). Not much of a surprise, the SP-chimera with the two BiP-dependent halves, APP/PrP, showed unchanged requirement for BiP in translocation of IDD-α2α3 (Fig. 2D, white panel; Fig. S2D). Although total hydrophobicity was elevated by their fusion, apolar residues still accumulated at the N-terminus and charged residues were lacking. Interestingly, PrP/Som-IDD-α2α3, the SP-chimera with a BiP-dependent N-terminus and a BiP-independent C-terminus, presented an intermediate phenotype (Fig. 2D, white panel; Fig. S2D). The hydrophobic Som-SP-C-terminus indeed led to a partial rescue; however we speculate that full capacity for BiP-independent translocation may require the basic residue at the N-terminus. Of note, the two complementing SP-chimera APP/Som- and PrP/APP-IDDα2α3 completely lost their capacity for translocation into the ER along with the loss of significant hydrophobicity of their SPs required for recognition by SRP and the Sec61 channel (Fig. S1A–C, Fig. S13) (Nilsson et al., 2015).

### Depletion of BiP differentially affects ER import of prion protein according to alpha-helical structures and the intrinsically disordered domain

Besides contribution of the SP to translocation efficiency, the alpha-helical domains at the C-terminus of the PrP were shown to promote translocation of its intrinsically disordered domain (IDD) (Miesbauer et al., 2009). We sought to clarify if this finding relates to the requirement for BiP that we had identified. To address the role of structural determinants in BiP-assisted translocation, another set of PrP variants with rearranged alpha-helices in the mature region (α2α3-IDD) were included into our study (Fig. 1C,F; Table S1) (Miesbauer et al., 2009). In fact, translocation efficiency of PrP- and APP-α2α3-IDD tremendously increased upon relocation of the helices towards the SP, thus, resembling BiP-independent translocation of Som-α2α3-IDD (Fig. 2D, yellow panel; Fig. S2E).

Considering that by switching the helices, the position of the IDD relative to the SP was likewise changed, we assumed that if presence of an IDD close to the SP affects PrP translocation, then its depletion would lead to loss of requirement for BiP similarly to its rearrangement. For this reason, translocation of IDD-depleted wt PrP (PrP-ΔIDD wt) was examined next as part of a third set of PrP variants (Fig. 1D,G). Unexpectedly, requirement for BiP significantly increased in the absence of the IDD (Fig. 2E, yellow panel; Fig. S2F). Thus, the IDD itself stimulated ER import or alternatively, the changing context of the mature region perturbed translocation, which was compensated by BiP. Hence, either the more distal position of the IDD allowed for more effective stimulation of translocation by the IDD or the phenotype of α2α3-IDD upon BiP depletion did not relate to changes of the IDD at all.

### Depletion of BiP inhibits ER import of prion protein due to a polybasic motif

BiP responded in a previous study on small presecretory proteins to a cluster of positively charged residues that is present in the downstream mature region of preproapelin (ppa) (Haßdenteufel et al., 2018). Interestingly, PrP likewise comprises an accumulation of basic amino acid residues, here, located adjacent to the SP. Despite detailed dissection of the functional domains in the PrP sequence, we note that previous investigations and some of the engineered PrP variants that had been investigated lacked this positively charged peptide (Davis et al., 2015; Hegde et al., 1998b; Kim and Hegde, 2002).

Consequently, we asked what impact this cluster of positively charged residues, hereafter called polybasic motif (KKRPK), has on the requirements of PrP for ER import. To address this question, we mutagenized all four basic residues of the polybasic motif to alanines (AAAPA) (Fig. 1A; Table S1). Strikingly, alanine substitution (A4) completely restored translocation of IDD-α2α3, even in the presence of a SP we had identified as BiP-dependent, such as PrP and APP (Fig. 2D, white panel; Fig. S2E). We further conclude that the remaining translocation apparatus is still functional and not affected by short treatment with subtilase toxin.

Similar observations were made for the mutagenized wt PrP, PrPA4 wt (Fig. 1D; Table S1). When the basic amino acid residues were replaced by alanines, PrP translocation into the ER of BiP depleted and control cells was equally efficient (Fig. 2E, white panel; Fig. S2). In sum, BiP-dependence of PrP translocation was reversed either by exchange of the SP or substitution of the polybasic motif.

Based on this finding, we considered that the altered position of these charges instead of the altered position of the helices may have accounted for the phenotype we have observed for α2α3-IDD. If so, re-insertion of the polybasic motif adjacent to the SP (PrPK4 and APPK4) would convert BiP-independent translocation into BiP-dependent (Fig. 1C; Table S1), despite the structural changes in α2α3-IDD. We found that K4-α2α3-IDD indeed phenocopied IDD-α2α3 which demonstrated that the polybasic motif only has a severe impact on translocation when it is located close to the SP (Fig. 2D, yellow panel; Fig. S2). We conclude that first, the altered translocation requirements of α2α3-IDD were caused by relocation of the charges rather than rearrangement of the alpha-helices and second, the effect of the charges decreases with increasing distance to the SP.

Convincingly, depletion of the polybasic motif was also effective in context of the IDD-depleted wt PrP (ΔK4-IDD) (Fig. 1D; Table S1), as the translocation defect shown by PrPΔIDD wt was partially relieved (Fig. 2E, yellow panel; Fig. S2F). Here, mutagenesis did not provide a full rescue of translocation efficiency. Thus, BiP did not have the capacity to compensate for both presence of the polybasic motif and depletion of the IDD.

### Depletion of Sec63 affects ER import of prion protein due to the signal peptide, alpha-helical structures, IDD and polybasic motif

Having identified multiple determinants for engagement of BiP in ER import of PrP, we continued evaluating whether contribution of the Hsp40-co-chaperone Sec63 is defined similarly (Lang et al., 2012). Following our established protocol, HeLa cells were treated with control or *SEC63* siRNA for 96 h (Table S3), before digitonin-permeabilization and analysis of the effects. As previously shown, cell growth and cell viability as well as other translocation components are not affected under these depletion conditions (Lang et al., 2012; Haßdenteufel et al., 2018). At a depletion efficiency of 95% (Fig. 2C; Fig. S2C), translocation was impaired accordingly to which signal peptide was preceding IDD-α2α3 (Fig. 2F, white panel; Fig. S2G). Congruent with the observations upon BiP depletion, the SPs derived of PrP and APP showed less translocation activity in the absence of Sec63 compared to the Som-SP. Transport of ppl and Cyt b5-OPG served as negative controls (Fig. 2F, blue panel; Fig. S2G,I).

On closer investigation, subtle differences emerged between the SP-specificity of Sec63 and BiP because the mixed SP-chimeras of IDD-α2α3 presented opposing phenotypes upon their depletion. Contrary to the intermediate phenotype of PrP/Som that we had been observed upon depletion of BiP, requirement for Sec63 remained unchanged and moreover, it was even strengthened in case of APP/PrP (c.f. Fig. 2D,F, white panels; Fig. S2G).

In line with this, translocation of Ppl-PrP wt still depended on the assistance of Sec63 though BiP-dependence was lost (c.f. Fig. 2E,G, white panels; Fig. S2I). We conclude that although engagement of Sec63 and BiP are dictated by the SP, each corresponds to different SP-characteristics.

Another non-correlating engagement of Sec63 and BiP may involve the IDD of wt PrP. In contrast to the persisting dependence on BiP (c.f. Fig. 2E,G, yellow panels; Fig. S2I), depletion of the IDD partially enabled Sec63-independent translocation of PrP wt. These data suggest that Sec63, additionally to its co-chaperone activity (see below), might have a specific function in translocation of the PrP-derived IDD, which involves an intrinsic activity.

Besides these differences between the phenotypes of Sec63 and BiP depletion, similar observations were made for the downstream polybasic motif. Its relocation or substitution led to alleviation of the requirement for Sec63 in translocation of all PrP-related variants used in this study, including IDD-α2α3, α2α3-IDD and PrP wt (Fig. 2F,G; Fig. S2H,I) and so was the effect of insertion of the polybasic motif visible in context of α2α3-IDD when translocated into the ER of Sec63-depleted HeLa cells (Fig. 2F,G; Fig. S2H). In sum, the charge-related engagement of Sec63 indeed correlated with the charge-related engagement of BiP.

Taken together, engagement of Sec63 and BiP in PrP translocation is dictated by distinct characteristics of the precursor polypeptide, hinting to multiple functions of both, either in collaboration or by themselves.

### Depletion of SR but not Sec62 or hSnd2 inhibits ER targeting of a PrP-related variant directed by the PrP-, APP- or Som-signal peptide

One may assume that the observed BiP-independent function of Sec63 is related to the supposed action as targeting receptor together with Sec62 (Davis et al., 2015; Lakkaraju et al., 2012). Regarding the growing complexity of the human ER targeting network (Casson et al., 2017; Haßdenteufel et al., 2018, 2017), we aimed to characterize the targeting route(s) taken by our set of PrP-related SP-chimera including the novel targeting factor hSnd2. Of note, IDD-α2α3 lacks potential targeting signals present within the PrP mature region, such as the TMD or GPI anchor sequence (Ast et al., 2013; Aviram et al., 2016; Davis et al., 2015; Hegde et al., 1998b).

**Fig. 3.**
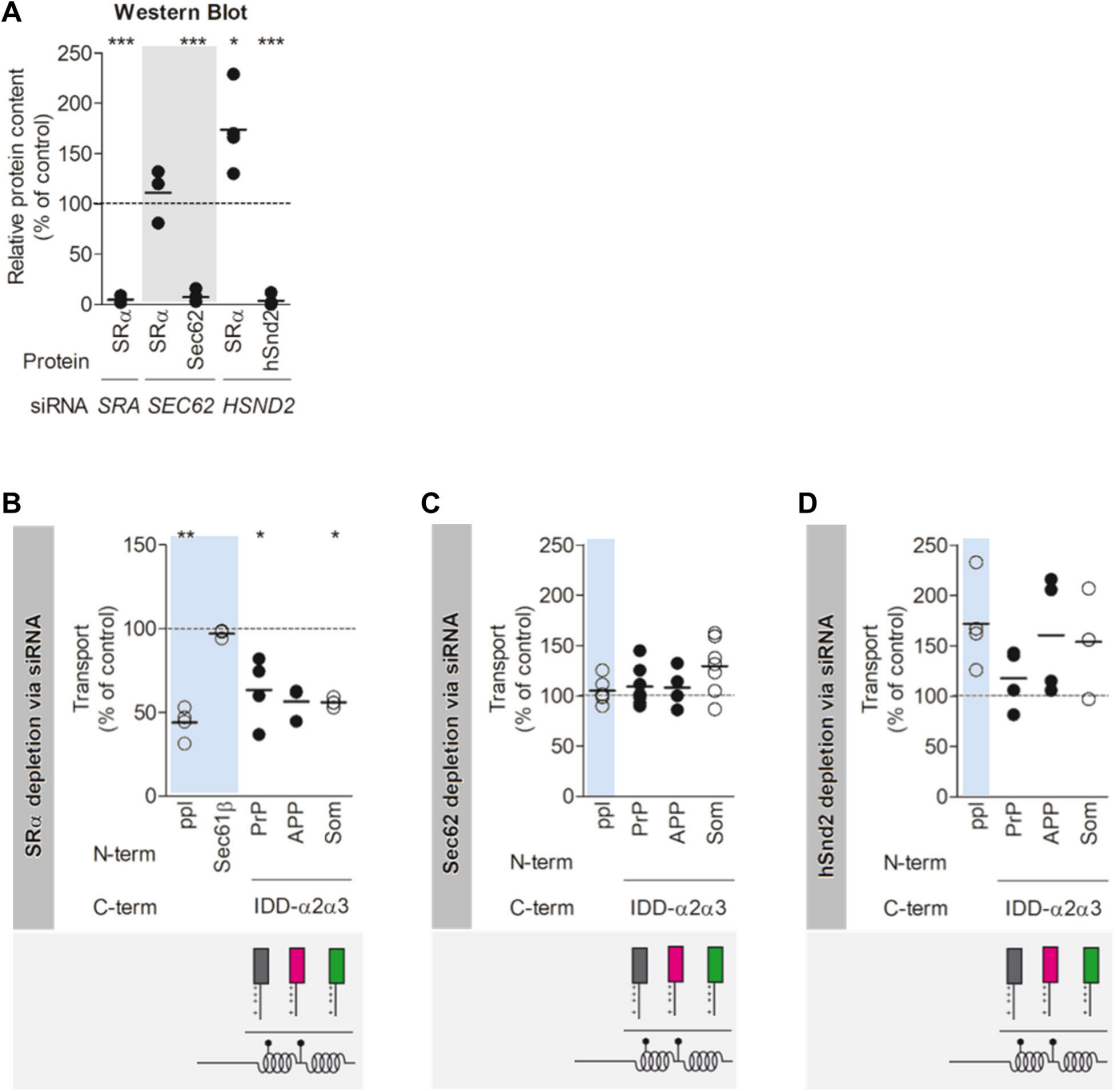
**Targeting of respective prion variants to the Sec61 complex mainly involves SR.** For protein depletion, HeLa cells were treated with the indicated siRNA (Table S3). After digitonin-permeabilization of the harvested cells (A–D), reticulocyte lysate was programmed with the indicated precursor polypeptides (ppl, Sec61β and IDD-α2α3 variants with various SPs) and incubated in the absence or presence of depleted or control ER membranes (B–D). Radioactive samples were subjected to SDS-PAGE and phosphorimaging (Fig. S3A,B). Transport efficiencies were calculated as the proportion of N-glycosylation or signal peptide cleavage of the total amount of synthesized precursors with the individual control sample set to 100%. Individual data points and the mean of at least three individual experiments are shown (D, *n*=2). For statistical analysis (***P<0.001, **P<0.01, *P<0.05), a Student's *t*-test was used. (A) Protein content of the indicated HeLa cells relative to β-actin was validated by western blot and corresponding antibodies (Fig. S3C,D). The control sample was set to 100%. (B) *SRA* siRNA effects. (C) *SEC62* siRNA effects. (D) *HSND2* siRNA effects. Filled dots, weak SPs; unfilled dots, strong SPs and controls; blue panel, control precursor polypeptides. See also Fig. S3. For complete phosphorimages, see Figs S11–S12.

To address how the different SPs target IDD-α2α3 to the ER membrane, we used established protocols for 96 h siRNA-based depletion of SR, Sec62 and hSnd2, respectively (Table S3) (Haßdenteufel et al., 2018; Johnson et al., 2013). As previously described, depletion of SR or hSnd2 results in a decline of cell survival (Haßdenteufel et al., 2017). Depletion of SR to 5% of the content in control cells strongly inhibited translocation of ppl (Fig. 3A,B; Fig. S3A,C) and consistently, compensatory upregulation of SR to 111–174% upon depletion of Sec62 or hSnd2 led to increased translocation efficiencies of ppl (Fig. 3A,C,D; Fig. S3A–D), as described before (Johnson et al., 2013; Haßdenteufel et al., 2017). Insertion of Sec61β-OPG remained unaffected in the absence of SR (Fig. 3B; Fig. S3A), as had to be expected (Haßdenteufel et al., 2017). Despite the varying hydrophobicity of the respective SPs (ΔG^pred^), all three PrP-related substrates showed strong preference for SR-dependent targeting, just as ppl (Figs 1B, 3B; Fig. S3A and Table S2). Closer inspection of the data revealed subtle differences. Upon hSnd2 depletion, the compensatory effect of upregulated SR was less pronounced on targeting by the PrP-SP and so it was upon Sec62 depletion, upregulation of SR less stimulating to targeting by the PrP- and APP-SP (Fig. 3C,D; Fig. S3A–B). Despite this potential underestimation of the effects, Sec62 and hSnd2 may only play minor roles in APP- and PrP-directed delivery of IDD-α2α3 to the ER in our experimental system. However, the SRP-SR route clearly dominated ER targeting of our PrP-related precursor polypeptides. We conclude that Sec63 and BiP were involved downstream of SR at a stage where protein targeting was completed.

## DISCUSSION

Several neurodegenerative diseases in humans and other mammals are linked to the PrP. Normally, it is attached to the external surface of the plasma membrane via a GPI-anchor. However, it shows an unusually complex topology, which is prone to aggregation and associated with pathogenesis of the diseases. Attributing its weak capacity for ER import to the causes, we investigated the requirements for targeting and translocation of the PrP precursor to the human ER. By defining rules for engagement of the auxiliary translocon components BiP and Sec63, some hitherto neglected determinants for the deficiency in translocation were identified.

### SR dominates in ER targeting directed by the PrP-, APP- or Som-signal peptide

We found clear domination of the SRP–SR route in targeting of our set of PrP-related precursor polypeptides to the human ER. Irrespectively of which SP was preceding IDD-α2α3, transport efficiencies of all variants decreased in the absence of SRα and tended to increase according to the compensatory overproduction of SR upon Sec62 or hSnd2 knockdown. However, targeting by the PrP- and APP-SP was less stimulated by the increased SRα content in the absence of Sec62 as it was for the PrP-SP in the absence of hSnd2. These observations might be attributed to minor roles of Sec62 and/or hSnd2 in transport of the respective variants. Incomplete gene silencing and resulting compensations on the protein level might have led to an underestimation of the depletion effects. We further note that by addressing a simplified PrP variant, IDD-α2α3, possible influence of additional targeting signals may have been ignored. A previous study showed impaired biogenesis of PrP upon complete Sec62 knockout in human cells which was interpreted as targeting phenotype since contribution of SRP-SR has not been observed (Davis et al., 2015). However, contribution of Sec62 has been fully attributed to the PrP-SP which is clearly not supported by the presented data here. The fact that only negligible effects were observed upon incomplete siRNA-mediated depletion is consistent with the view of a regulatory function of Sec62 in Sec61 gating and excludes a function as main targeting receptor together with Sec63 (Haßdenteufel et al., 2018; Lang et al., 2012). Late recruitment of Sec62 and Sec63 to the translocon also argues against a role of the two proteins in PrP targeting (Conti et al., 2015).

Regarding putative hSnd2-mediated targeting of the mouse GPI-anchored PrP precursor, the PrP-SP might principally have the capacity for entry into the hSnd-pathway. The absence of additional internal or C-terminal signals may have reduced efficiency of recognition accordingly to what has been described in yeast (Ast et al., 2013; Aviram et al., 2016). However, clear determinants for this route have not yet been defined in human (Haßdenteufel et al., 2017).

In sum, the investigated signal peptides were hydrophobic enough for recognition by SRP and so, they differed only marginally with respect to their capacity for targeting to the Sec61 complex, which is independent of their capacity for channel gating.

### BiP and its co-chaperone Sec63 mediate Sec61 channel gating in the case of prion protein precursors with weak signal peptides in combination with detrimental mature regions

**Fig. 4.**
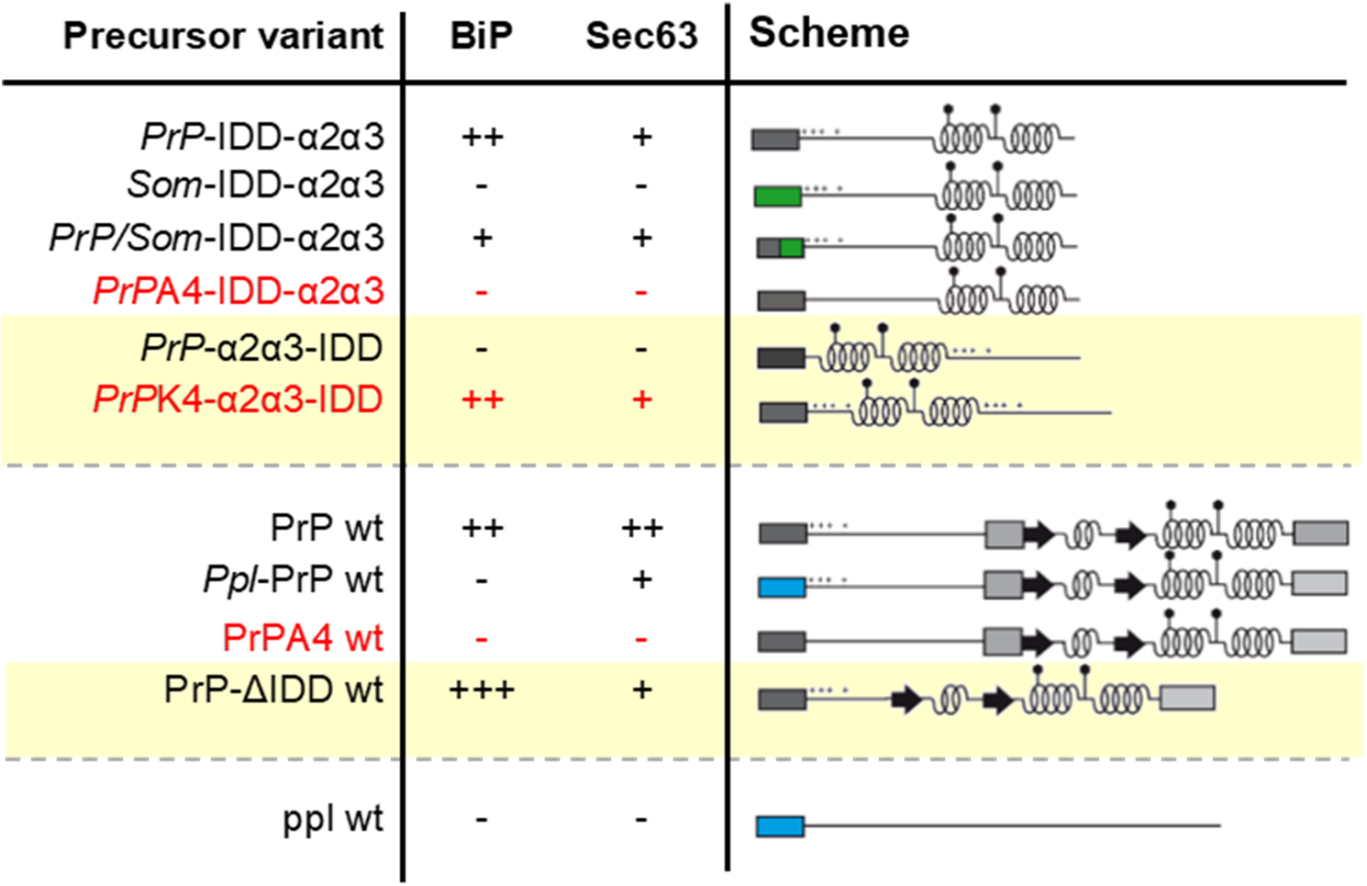
**Summary of the observed requirements in ER import of key precursor proteins.** Summarized are the key results for dependency on BiP and Sec63 of the investigated model precursor proteins (see Fig. 2). +++, super strongly dependent; ++, strongly dependent; +, dependent; −, independent.

The presented study revealed that BiP-dependence in translocation of the PrP precursor is determined by a combination of both a weak N-terminal signal peptide and a detrimental region in the adjacent mature part. The SPs derived of the PrP and APP precursors were characterized as BiP-dependent in contrast to the SPs derived of the Som precursor and ppl. While all four SPs showed basic capacity for SRP-SR mediated targeting to the ER, they seem to differ in their capacity for Sec61 channel gating (Fig. 4). Our set of PrP-related SP-chimeras allowed us to define the weakness as a function of apolar and basic amino acid residues distributed along the N-terminus of the precursor polypeptide. It includes the SP and the early mature region where we found a previously unappreciated polybasic motif. Respective characteristics may dictate the mode of insertion, i.e. orientation and dwell time of the SP within the channel, and the capacity for intercalation of the SP into the lateral gate equivalent to displacement of Sec61-helix 2 (Voorhees and Hegde, 2016).

**Fig. 5.**
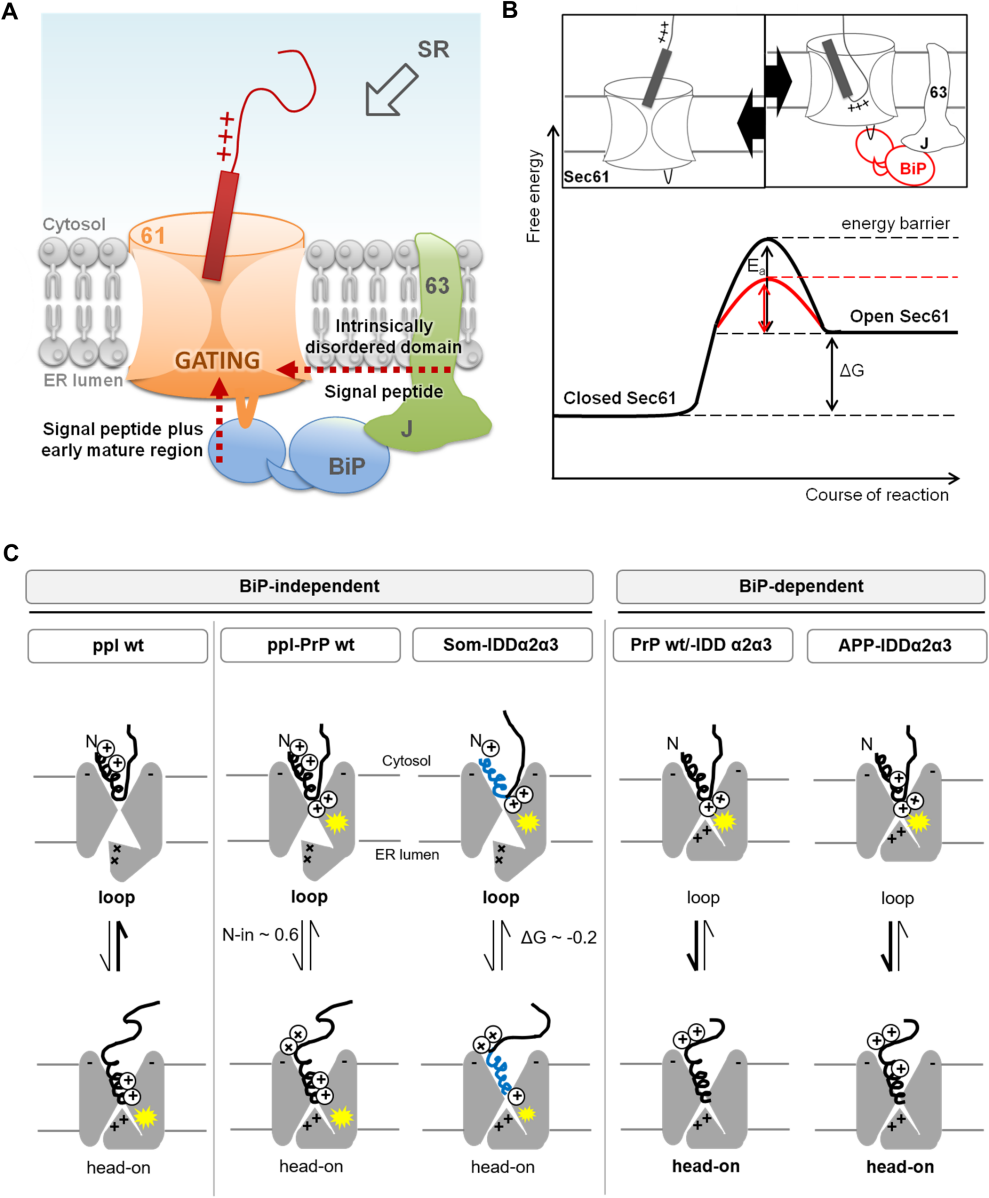
**Model for chaperone-mediated Sec61 gating in prion protein transport.** (A) Unproductive sampling of the Sec61 interior and head-on insertion of precursor polypeptides with polar and hydrophilic SPs in combination with detrimental features in the mature region (PrP, red) may cause a delay in translocation and recruitment of the auxiliary translocon components BiP and Sec63. They facilitate Sec61 channel opening either as co-chaperone/chaperone-pair or by an intrinsic activity (Sec63) and so, compensate for the weakness of the SP, cluster of positive charges adjacent to the SP and intrinsically disordered domains. (B) Energy diagram for BiP-mediated Sec61 channel gating (red line) and model for chaperone-assisted loop-insertion of the precursor polypeptide into Sec61 (right cartoon). In the case of original head-on insertion (left cartoon) (Fig. S3), they may not provide the energy required for transition from the closed towards the open state by themselves (black line). (C) Orientation of the inserted polypeptide within the Sec61 complex (grey) is regulated by charges in the precursors as well as the channel, i.e. cytosolic loops and plug domain (triangle-shaped hinge). For channel opening, the tip of the SP (spiral) is typically exposed in the cytosol with the polypeptide chain forming a loop-structure oriented towards the ER lumen. A polybasic motif (pluses) in the early mature region of PrP clashes (yellow star) with respective positive charges in the plug domain and therefore, interferes with loop-insertion. The BiP-independent SPs derived of ppl and Som provide positively charged amino acid residues at the amino-terminus (N) which compensate for the imbalance of charges and so, precursor insertion switches from head-on to loop. In addition, distribution of apolar residues (blue) along the SP and total hydrophobicity (ΔG^pred^) may influence the mode of insertion, i.e. loop or head-on, which might be reflected as well by N-in^pred^. wt, wild type. Cartoon design based on Junne et al., 2007.

Either exchange of the SP or deletion and relocation of the polybasic motif reversed requirement for BiP in PrP translocation. Therefore, we propose that the polybasic motif in the early mature region amplifies weakness of the preceding SP and so interferes with insertion into the Sec61 channel. Like the positive-inside rule for topology determination of membrane proteins, the polybasic motif may favor head-on insertion instead of the typical loop-insertion of soluble precursor polypeptides (Fig. 5A,C). Since loop-insertion is supposed to be a prerequisite for Sec61 gating, we suggest that BiP must assist by binding to ER luminal loop 7 of the Sec61 alpha-subunit to compensate for the imbalance of charges in the early precursor polypeptide (Lang et al., 2012, 2017; Schäuble et al., 2012). Alternatively, BiP may be required for the flip-turn of SP after initial head-on insertion. Likewise, positively charged amino acid residues at the N-terminus of a SP may compensate for respective charges at its C-terminus and provoke spontaneous loop-formation without assistance by BiP. SPs which meet this requirement, such as Som- and ppl-, therefore enabled translocation even in the presence of the detrimental mature region and in the absence of BiP.

The mixed SP-chimera PrP/Som-IDD-α2α3 additionally suggested that the distribution of hydrophobic amino acid residues along the signal peptide influences orientation of the inserted precursor within the pore, as proposed for membrane proteins (Harley et al., 1998). The hydrophobic profile of the SP may further determine its capacity for intercalation into the lateral gate set by ‘placeholder’ helix 2 (Voorhees and Hegde, 2016). In sum, the N-terminal cleavable SP of soluble precursor proteins follows the same rules for insertion into the lateral gate as membrane proteins do for integration into the ER membrane.

We note that an influence of N-terminal charges on translocation initiation and PrP topology has been previously described (Kim and Hegde, 2002; Nilsson et al., 2015). The presented data now link compensation for the loss of charges in the PrP signal peptide to BiP action at the translocon.

Our previous studies on translocation of small presecretory proteins identified the SP and the detrimental mature region as exclusive determinants for engagement of BiP (Haßdenteufel et al., 2018; Johnson et al., 2013). Distinctive to the PrP-derived polybasic motif described here, the cluster of positively charged residues in ppa is located more downstream in the mature region and still effective in context of a strong SP, such as of ppl. Based on this discrepancy, we assume that the clustered charges in PrP and ppa differentially affect precursor insertion into the Sec61 channel, possibly depending on the different dwell times of the two SP at the cytosolic face of the Sec61 channel.

Firstly, the inhibitory effect depended on the distance between the charges and the respective SP. Consistently, rearrangement of helices in the PrP sequence and subsequent relocation of the charges towards the C-terminus drastically diminished their impact on translocation in case of the PrP precursor. These data further support the view that the early mature region and the SP build a functional unit which is structurally reflected by its capacity for loop-formation (Alken et al., 2009). Here, we extend this model by auxiliary translocon component BiP in the case of an ineffective pairing of mature region and SP.

Secondly, the inhibitory effect depended on the context of the charges. Regardless of the distance, the ppl-SP indeed enabled BiP-independent translocation of a basic cluster in context of the PrP but it did not in context of the parental protein or apelin (Nilsson et al., 2015). We assume that the capacity for compensation by the SP relies on the mode of inhibition, which does not necessarily depend on the localization of the charges. However, the combination of a detrimental region with a weak SP may provide more time for channel gating and/or recruitment of auxiliary translocon components (Conti et al., 2015; Kang et al., 2006).

Alternatively, these contradictory data reflect context-dependence with respect to the overall length of the precursor polypeptide. According to the concept of dwell time for Sec61 gating (Zhang and Miller, 2012), small precursor polypeptides have less time for opening of the channel which might make them prone for detrimental charges in the mature region. Remarkably, in both cases, the charges are implemented in an intrinsically disordered domain. However, the inhibitory effect of the polybasic motif was still observed in context of the IDD-depleted PrP variant.

Nevertheless, IDD-depletion alone impaired translocation of the wt PrP in the absence of BiP, most likely due to similar kinetic reasons. Presence of the IDD as spacer between the SP and TMD may provide the SP more time to gate the Sec61 channel on its own and thus, attenuate requirement for BiP (Kim and Hegde, 2002). Alternatively, the IDD may even have gating capacity by itself (Alken et al., 2009). Either way, BiP compensated for the loss of the IDD or the resultant contextual changes and lastly for the weak gating capacity of the PrP-SP.

Despite the proposed mechanistical differences between the two types of basic clusters found in PrP and ppa, both resulted in the same requirement for BiP in Sec61 gating. BiP-mediated channel opening might be best explained by an energy-driven working model analogous to an enzyme-catalyzed reaction (Fig. 5B) (Haßdenteufel et al., 2018). In doing so, BiP may facilitate flip-turn of head-on inserted PrP polypeptides and intercalation of its weak SP into the lateral gate (Devaraneni et al., 2011). Based on the correlating phenotypes upon changes to the polybasic motif, we further assume that BiP is supported by the Hsp40-co-chaperone Sec63 as it is in translocation of small presecretory proteins (Fig. 5A). We note that the effects upon Sec63 depletion were less pronounced compared to the effects observed upon BiP depletion, which we assume was caused by the different depletion efficiencies of the approaches used.

### Sec63 by itself mediates Sec61 channel gating in the case of prion protein precursors with weak signal peptides or intrinsically disordered domains

Our pre-designed set of precursor polypeptides allowed us to dissect multiple implications of Sec63 in PrP transport. Not all of them seemed related to its established role as Hsp40-co-chaperone in BiP-mediated Sec61 opening (Haßdenteufel et al., 2018; Schorr et al., 2015), because it responded differently than BiP to changes of the SP and IDD (Figs 4, 5A). Although Sec63 and BiP were selectively engaged in translocation of the same SPs, i.e. PrP and APP, opposite phenotypes of the mixed SP-chimeras also suggested different rules for engagement of each component. These data argue for an additional function of Sec63 which is not associated with BiP nor with Sec62, as discussed above. Such intrinsic activity of Sec63 was appreciated before in membrane integration of aquaporin 2 and invariant chain and translocation of the small presecretory proteins ppa and prestatherin (Haßdenteufel et al., 2018; Lang et al., 2012). However, the ppl-SP was not able to provide capacity for Sec63-independent translocation in context of the PrP opposing to our previous observations. Which SP-properties lastly determined requirement for intrinsic Sec63 activity in PrP transport was difficult to reveal by the addressed variants here and under the terms of multiple Sec63 functions.

Interestingly, we observed the tendency that Sec63 is specifically engaged in translocation also of IDDs as its deletion reduced requirement for Sec63 but not BiP (Fig. 4). Previous studies demonstrated deficiency of the Sec61 channel in translocation of IDDs which is promoted by alpha-helices in the precursor sequence (Dirndorfer et al., 2013; Gonsberg et al., 2017; Jung and Tatzelt, 2018; Miesbauer et al., 2009). In addition to stabilizing alpha-helical structures, Sec63 may compensate for this deficiency by assisting flexible polypeptides to insert into the Sec61 channel (Fig. 5A).

In summary, the present study examined the PrP and distinct N-terminal cleavable signal peptides with respect to their requirements for ER import. Besides similar capacities for ER targeting, they showed different capacities for translocation which is compensated by the auxiliary translocon components BiP and Sec63. Chaperone-mediated Sec61 gating involves Sec63 assisted binding of BiP to loop 7 of the Sec61 alpha-subunit and an intrinsic activity of Sec63. Engagement of BiP in PrP transport is dictated by the distribution of basic and apolar amino acid residues at the N-terminus of the precursor polypeptide. Interestingly, the presented polybasic motif in the early mature region which amplified weakness of the preceding SP plays a crucial role as toxic effector domain in the mature protein (Wu et al., 2017). After identification of a positively charged cluster in the downstream sequence of preproapelin, here we present a second example of a functional mature domain which most likely interferes with loop-insertion of the precursor polypeptide into the Sec61 channel. We further conclude that BiP reduces prion pathogenesis at two stages, firstly, during early biogenesis and topology determination at the translocon and secondly, in propagation of disease-associated PrP^Sc^ by direct binding to the polypeptide chain (Park et al., 2017).

## MATERIALS AND METHODS

### Cloning and site-directed mutagenesis

The signal peptide region of PrP wt was replaced by the region coding for the ppl signal peptide, according to standard cloning procedures. Site-directed mutagenesis was used for deletion, alanine substitution (AAAPA) or insertion, respectively, of a polybasic motif (KKRPK) in the early mature region of PrP. For the other plasmids encoding mouse PrP variants, see Pfeiffer et al. (2013).

### Cell culture

HeLa cells (DSM no. ACC 57) were obtained from the German Collection of Microorganisms and Cell Cultures, routinely tested for mycoplasma contamination by VenorGeM mycoplasm Detection Kit (Biochrom/Merck, Berlin, Germany), and replaced every 5 years by a new batch. Cells were cultivated at 37°C in Dulbecco's modified Eagle's medium (DMEM; Gibco/Thermo Fisher Scientific, Bonn, Germany) containing 10% foetal bovine serum (FBS; Biochrom, Berlin, Germany) and 1% penicillin/streptomycin (GE Healthcare, Freiburg, Germany) in a humidified environment with a 5% CO_2_ atmosphere. Cell growth was monitored using the Countess^®^ Automated Cell Counter (Invitrogen/Thermo Fisher Scientific) according to the manufacturer's instructions.

### Depletion of cells by siRNA or toxin treatment

For manipulation, 5.2×10^5^ HeLa cells were seeded in a 6-cm culture plate in normal culture. For gene silencing, HeLa cells were transfected with targeting or control siRNA (Table S3) (Applied biosystems/Thermo Fisher Scientific and Qiagen, Hilden, Germany) to a final concentration of 15–35 nM using HiPerFect Reagent (Qiagen) as described previously (Haßdenteufel et al., 2018; Johnson et al., 2013; Lang et al., 2012). The cells were transfected a second time after 24 h with fresh medium. Western blotting was used to evaluate silencing efficiencies with the help of respective rabbit antibodies and a mouse anti-β-actin antibody (1:1000 dilution) (A5441, Sigma/Merck, Darmstadt, Germany). Rabbit antibodies were raised against the C-terminal peptides of human SRα (10-mer; 1:200 dilution), Sec62 (11-mer; 1:1000 dilution), Sec63 (13-mer; 1:500 dilution); the amino terminal peptides of human BiP (12-mer; 1:500 dilution); the C-terminal peptide of human hSnd2 (14-mer; 1:250 dilution) (Haßdenteufel et al., 2018). The primary antibodies were visualized using goat anti-rabbit IgG-peroxidase conjugate and ECL™ (1:1000 dilution) (A8275, Sigma/Merck, Darmstadt, Germany), ECL™ Plex goat anti-rabbit IgG-Cy5 (1:1000 dilution) or ECL™ Plex goat anti-mouse IgG-Cy3 conjugate (1:2500 dilution) (PA45011 and PA43009, GE Healthcare), and the Fusion SL (Peqlab, Erlangen, Germany) luminescence imaging system or the Typhoon-Trio imaging system in combination with Image Quant TL 7.0 software (GE Healthcare). For alternative strategy of BiP depletion, HeLa cells were treated with the subtilase cytotoxin SubAB or the inactive mutant SubA_A272_B at a final concentration of 1 µg/ml for 2 h (Paton et al., 2006; Schäuble et al., 2012).

### Protein transport

Precursor polypeptides (Table S1) were synthesized in reticulocyte lysate (nuclease treated; Promega, Heidelberg, Germany) in the presence of [^35^S]methionine (Perkin Elmer, Rodgau-Jügesheim, Germany) and buffer or semi-permeabilized cells (final concentration: 6400 cell equivalents/µl for human Sec61β-OPG (Johnson et al., 2013) and 12,800 equivalents/µl for mouse PrP variants and bovine ppl (Schlenstedt et al., 1990)) for 60 min at 30°C (co-translational transport). In case of the tail-anchored protein Cyt b5-OPG (Lang et al., 2012), reticulocyte lysate was first programmed and incubated with [^35^S]methionine for 15 min at 30°C. Before adding buffer or semi-permeabilized cells (final concentration: 6400 cell equivalents/µl) and incubation for another 20 min at 30°C, incubation continued for 5 min at 30°C in the presence of puromycin (final concentration: 1 mM). The cells were pre-treated with targeting or control siRNA for 48-96 h. Digitonin-permeabilized cells were prepared from equal cell numbers according to the published procedure (Haßdenteufel et al., 2018; Johnson et al., 2013; Lang et al., 2012). Following translocation, membranes were re-isolated by centrifugation at 125,000× ***g*** at 4°C for 20 min when required. For demonstration of N-glycosylation, the translocation reaction was co-translationally incubated in the presence of the tripeptide NYT (final concentration: 0.1 mM) or H_2_O, where indicated. All samples were analyzed by SDS-PAGE and phosphorimaging (Typhoon-Trio imaging system). Image Quant TL 7.0 was used for quantifications. Silencing efficiency was evaluated by western blot.

### Graphical representation and statistical analysis

Dot plots depict relative transport efficiencies calculated as the proportion of N-glycosylation or signal peptide cleavage of the total amount of synthesized precursors with the individual control sample set to 100%. Data points and the mean of at least three individual experiments were visualized with GraphPad Prism 5 software. A two-tailed Student's *t*-test was used for statistical comparison between a treatment group and the corresponding control (indicated by the upper panel). ANOVA in combination with the Dunnett's (wt set as control sample) and Newman–Keuls post hoc test, respectively, were performed on normalized values to compare between multiple precursor variants (indicated by horizontal brackets). Significance levels are given as follows: ***P<0.001, **P<0.01, *P<0.05.

## Supplementary Material

Supplementary information
