# Supplementary material for: The signal peptide plus a cluster of positive charges in prion protein dictate chaperone-mediated Sec61 channel gating
Source: Biol Open. 2019 Feb 11;8(3):bio040691. doi: 10.1242/bio.040691 (PMC6451349; doi:10.1242/bio.040691)
Supplement: Supplementary information [file biolopen-8-040691-s1.pdf]

Table S1: Amino acid sequences of the model precursor proteins and signal peptides, Related to Fig. 1.

Underlined: SP or TMD; underscore: fusion area in mixed SPs; italics/blue: IDD; bold: α-helical regions 2 and 3; red: positively charged amino acid residues of interest;

Name	Sequence
PrP-SP (mouse)	MANLGYWLLALFVTMWTDVGLC
APP-SP (human)	MLPGLALLLLAAWTARALEVPTDG
Som-SP (human)	MLSCRLQCALAALSIVLALGCVTG
APP/PrP-SP	MLPGLALLLLA_LFVTMWTDVGLC
PrP/Som-SP	MANLGYWLLA_LSIVLALGCVTG
ppl-SP (bovine)	MDSKGSSQKGSRLLLLLVVSNLLLCQGVVS
IDD-α2α3	KKRPKPGGWNT*GGSRYPGQGSPGGNRYPPQGGTWGQPHGGGWGQPH*<br>*GGSWGQPHGGSWGQPHGGGWGQGGGTHNQWNKPSKPKTNLKHVAGA*<br>**QNNFVHDCVNITIKQHTVTTTT**KGENFT**ETDVKMMERVVEQMCVTQYQKE**
α2α3-IDD	**QNNFVHDCVNITIKQHTVTTTT**KGENFT**ETDVKMMERVVEQMCVTQYQKE**<br>KKRPKPGGWNT*GGSRYPGQGSPGGNRYPPQGGTWGQPHGGGWGQPH*<br>*GGSWGQPHGGSWGQPHGGGWGQGGGTHNQWNKPSKPKTNLKHVAGA*
A4-IDD-α2α3	AAAPAPGGWNT*GGSRYPGQGSPGGNRYPPQGGTWGQPHGGGWGQPH*<br>*GGSWGQPHGGSWGQPHGGGWGQGGGTHNQWNKPSKPKTNLKHVAGA*<br>**QNNFVHDCVNITIKQHTVTTTT**KGENFT**ETDVKMMERVVEQMCVTQYQKE**
K4-α2α3-IDD	KKRPK**QNNFVHDCVNITIKQHTVTTTT**KGENFT**ETDVKMMERVVEQMCVTQ**<br>**YQKE**KKRPKPGGWNT*GGSRYPGQGSPGGNRYPPQGGTWGQPHGGGWG*<br>*QPHGGSWGQPHGGSWGQPHGGGWGQGGGTHNQWNKPSKPKTNLKHV*<br>*AGA*
PrP wt (mouse)	MANLGYWLLALFVTMWTDVGLC<br>KKRPKPGGWNT*GGSRYPGQGSPGGNRYPPQGGTWGQPHGGGWGQPH*<br>*GGSWGQPHGGSWGQPHGGGWGQGGGTHNQWNKPSKPKTNLKHVAGA*<br>*AAAGAVV*

	GGLGGYMLGSAMSRPMIHFGNDWEDRYYRENMYRYPNQVYYRPVDQYSN<br>**QNNFVHDCVNITIKQHTVTTTT**KGENFT**ETDVKMMERVVEQMCVTQYQKE**S<br>QAYYDGRRSSSTVLFSSPPVILLISFLIFLIVG
PrPΔIDD wt	MANLGYWLLALFVTMWTDVGLC<br>KKRPKPGGWNT<br>GGLGGYMLGSAMSRPMIHFGNDWEDRYYRENMYRYPNQVYYRPVDQYSN<br>**QNNFVHDCVNITIKQHTVTTTT**KGENFT**ETDVKMMERVVEQMCVTQYQKE**S<br>QAYYDGRRSSSTVLFSSPPVILLISFLIFLIVG
PrPΔK4-IDD wt	MANLGYWLLALFVTMWTDVGLC<br>GGLGGYMLGSAMSRPMIHFGNDWEDRYYRENMYRYPNQVYYRPVDQYSN<br>**QNNFVHDCVNITIKQHTVTTTT**KGENFT**ETDVKMMERVVEQMCVTQYQKE**S<br>QAYYDGRRSSSTVLFSSPPVILLISFLIFLIVG
ppl wt (bovine)	MDSKGSSQKGSRLLLLLVVSNLLLCQGVVS<br>TPVCPNGPGNCQVSLRDLFDRAVMVSHYIHDLSSEMFNEFDKRYAQGKGFI<br>TMALNSCHTSSLPTPEDKEQAQQTHHEVLMSLILGLLRSWNDPLYHLVTEV<br>RGMKGAPDAILSRAIEIEEENKRLLEGMEMIFGQVIPGAKETEPYPVWSGLP<br>SLQTKDEDARYSAFYNLLHCLRRDSSKIDTYLKLLNCRIIYNNNC

Table S2: Characteristics of the model signal peptides, Related to Fig. 1.

ΔG^{pred}: ΔG prediction by ΔG prediction server v1.0 (http://dgpred.cbr.su.se); N-in^{pred}: N-in prediction by TMHMM Server v. 2.0 (http://www.cbs.dtu.dk/services/TMHMM/).

Signal peptide	Length (amino acids)	Charges (SP)	ΔG^{pred} (SP)	N-in^{pred} (SP)
PrP-	22	0	0.63	0.27
APP-	24	1+	0.02	0.01
Som-	24	1+	-0.19	0.36
APP/PrP-	23	0	-1.24	0.29
PrP/Som-	22	0	-0.89	0.16
ppl-	30	3+/-	5.36	0.56

Table S3: Sequences of siRNAs used in this study, Related to Materials and Methods.

siRNA name	Target gene	Target sequence	Source	Concen-tration (nM)	Time (h)
BIP	*HSPA5*	AAGCGGCTGTTTACTGCTTTT	Qiagen	35	48
SEC62	*SEC62*	AAGGCTGTGGCCAAGTATCTT	Applied	15	96
SEC63	*SEC63*	AAGGGAGGTGTAGTTTTTTTA	Applied	15	96
HSND2	*TMEM208*	CACCTTAAGGATGTGATCCTA	Qiagen	20	96
SRA	*SRPRA*	CACCAGAGCTTTGCTAATAAT	Qiagen	15	96

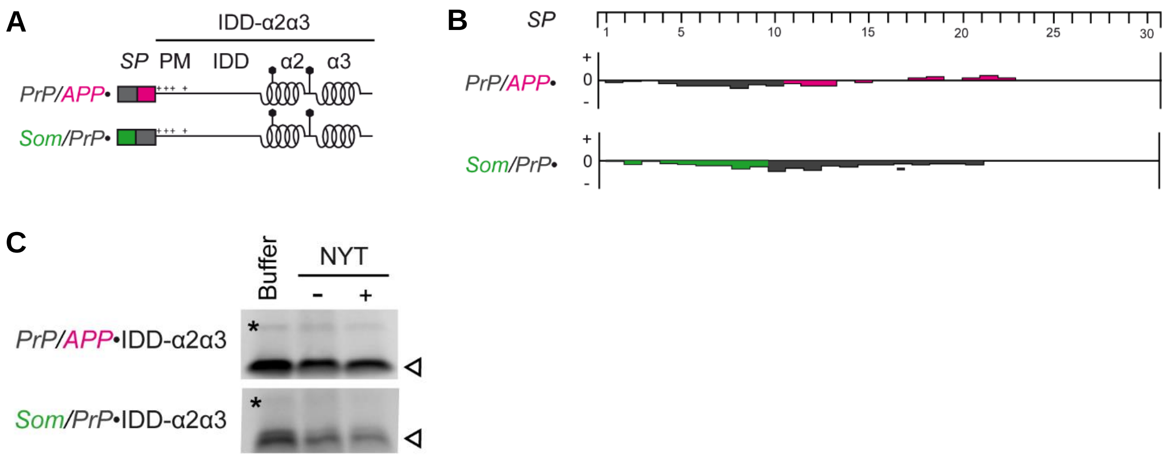

Fig. S1. Loss of translocation capacity in model precursor proteins with chimeric signal peptides, Related to Fig. 1.

(**A**) Schematic representation of two IDD-α2α3 variants with chimeric signal peptides composed of the N-terminal half of either PrP or Som and the C-terminal half of either APP (PrP/APP) or PrP (Som/PrP) (complementing the chimeric constructs shown in Fig. 1A,B,E). SP, signal peptide (italics); PM, polybasic motif (pluses); IDD, intrinsically disordered domain; α2α3, α-helical regions 2 and 3; β, beta-sheet; lollipops, N-glycans. (**B**) Kyte-Doolittle Hydrophobicity Plots of the chimeric signal peptides. Charged amino acid residues are indicated. Scale: -4.5 to +4.5. PrP/APP-SP: MANLGYWLLA_AWTARALEVPTDG (ΔG^{pred}: 3.96); Som/PrP-SP: MLSCRLQCA_LFVTMWTDVGLC (ΔG^{pred}: 1.79); ΔG^{pred}, ΔG prediction server v1.0 (http://dgpred.cbr.su.se). (**C**) PrP/APP-IDD-α2α3 and Som/PrP-IDD-α2α3 lack capacity for ER translocation. The indicated PrP variants were synthesized in reticulocyte lysate in the absence (i.e. presence of buffer) or presence of membranes and the tripeptide NYT, respectively. All samples were subjected to SDS-PAGE and phosphorimaging. Relevant parts of the phosphorimages are shown. unfilled triangle: precursor polypeptide; star, putatively ubiquitinated precursor polypeptide (Rane et al., 2008); PrP, prion protein; APP, amyloid precursor protein; Som, somatostatin; wt, wild type. For complete phosphorimages, see Fig. S13.

Biology Open • Supplementary information

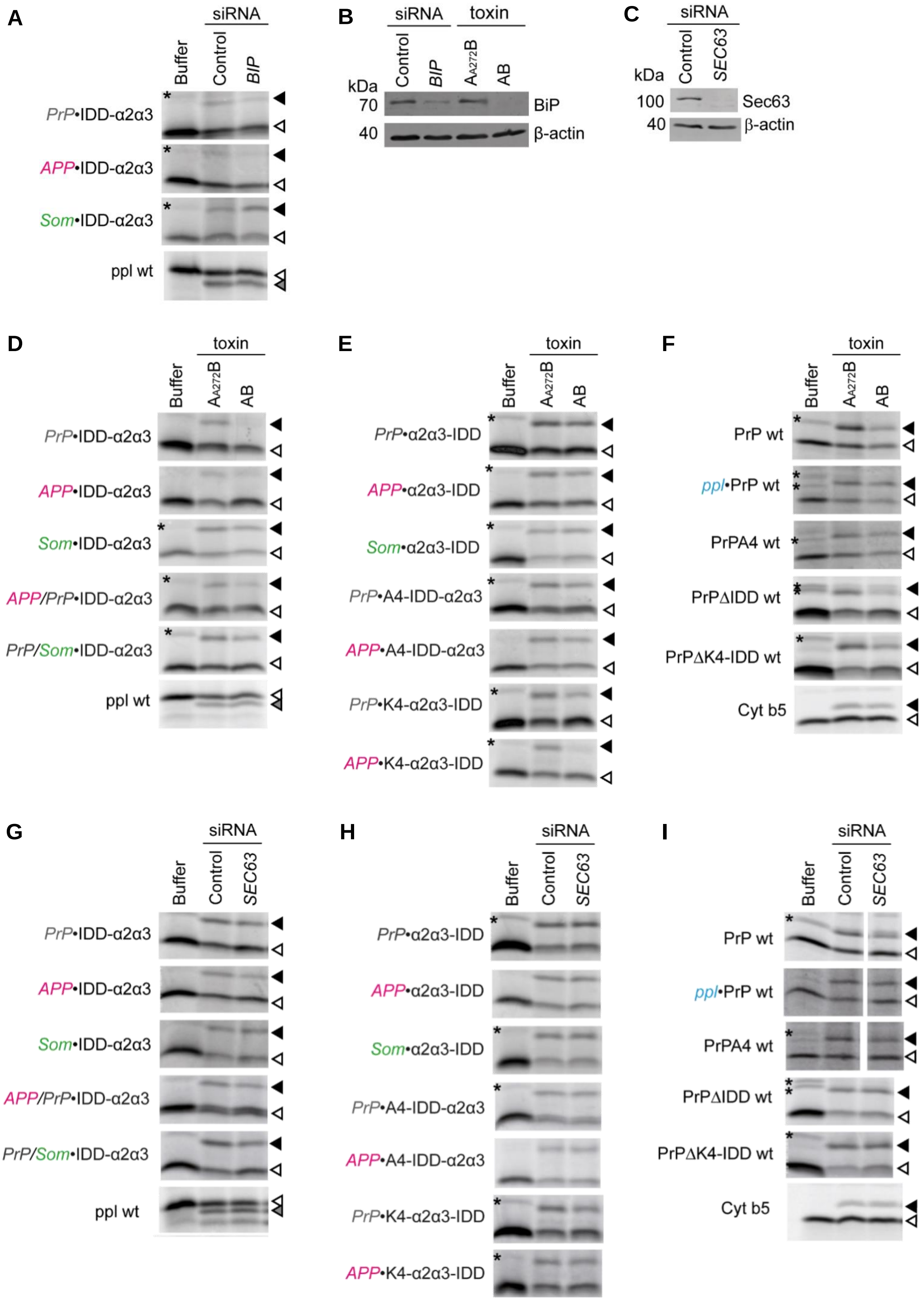
A
siRNA
Buffer
Control
BIP
PrP•IDD-α2α3
APP•IDD-α2α3
Som•IDD-α2α3
ppl wt
B
siRNA
toxin
Control
BIP
AA272B
AB
kDa
70
40
BiP
β-actin
C
siRNA
kDa
Control
SEC63
100
40
Sec63
β-actin
D
toxin
Buffer
AA272B
AB
PrP•IDD-α2α3
APP•IDD-α2α3
Som•IDD-α2α3
APP/PrP•IDD-α2α3
PrP/Som•IDD-α2α3
ppl wt
E
toxin
Buffer
AA272B
AB
PrP•α2α3-IDD
APP•α2α3-IDD
Som•α2α3-IDD
PrP•A4-IDD-α2α3
APP•A4-IDD-α2α3
PrP•K4-α2α3-IDD
APP•K4-α2α3-IDD
F
toxin
Buffer
AA272B
AB
PrP wt
ppl•PrP wt
PrPA4 wt
PrPΔIDD wt
PrPΔK4-IDD wt
Cyt b5
G
siRNA
Buffer
Control
SEC63
PrP•IDD-α2α3
APP•IDD-α2α3
Som•IDD-α2α3
APP/PrP•IDD-α2α3
PrP/Som•IDD-α2α3
ppl wt
H
siRNA
Buffer
Control
SEC63
PrP•α2α3-IDD
APP•α2α3-IDD
Som•α2α3-IDD
PrP•A4-IDD-α2α3
APP•A4-IDD-α2α3
PrP•K4-α2α3-IDD
APP•K4-α2α3-IDD
I
siRNA
Buffer
Control
SEC63
PrP wt
ppl•PrP wt
PrPA4 wt
PrPΔIDD wt
PrPΔK4-IDD wt
Cyt b5

Fig. S2. Effects of *BiP* and *SEC63* siRNA and subtilase toxin on transport of prion protein precursor variants, Related to Fig. 2.

(**A,D-I**) Phosphorimages of representative SDS-PAGE gels. After treatment with the indicated siRNA (A,**G-I**) or subtilase toxin (**D-F**), HeLa cells were digitonin-permeabilized and used for *in vitro* transport of the indicated precursor polypeptides into the ER in the presence of reticulocyte lysate. (**B,C**) Representative Western Blots validating protein content. filled triangle, glycosylated protein; unfilled triangle: precursor polypeptide; star, ubiquitinated precursor polypeptide. For complete phosphorimages and Western Blots, see Figs. S6-10.

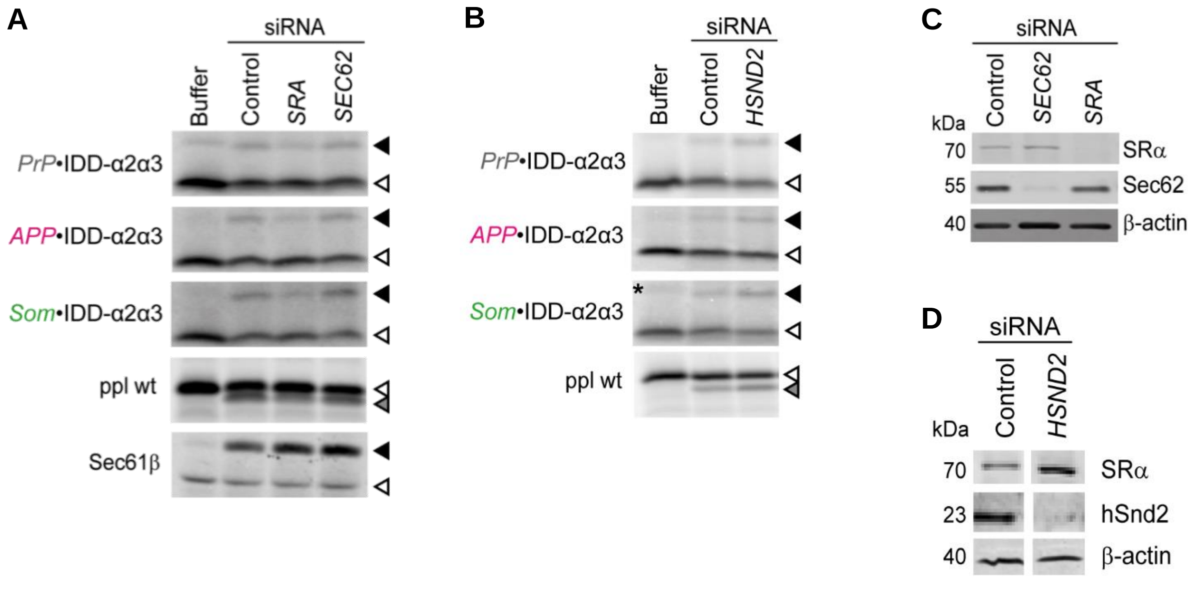

Fig. S3. Effects of *SRA, SEC62* and *HSND2* siRNA on transport of prion protein precursor variants, Related to Fig. 3.

(**A,B**) Phosphorimages of representative SDS-PAGE gels. After treatment with the indicated siRNA, HeLa cells were digitonin-permeabilized and used for *in vitro* transport of the indicated precursor polypeptides into the ER in the presence of reticulocyte lysate. (**C,D**) Representative Western Blots validating protein content. filled triangle, glycosylated protein; unfilled triangle: precursor polypeptide. For complete phosphorimages and Western Blots, see Figs. S11, S12.

Biology Open • Supplementary information

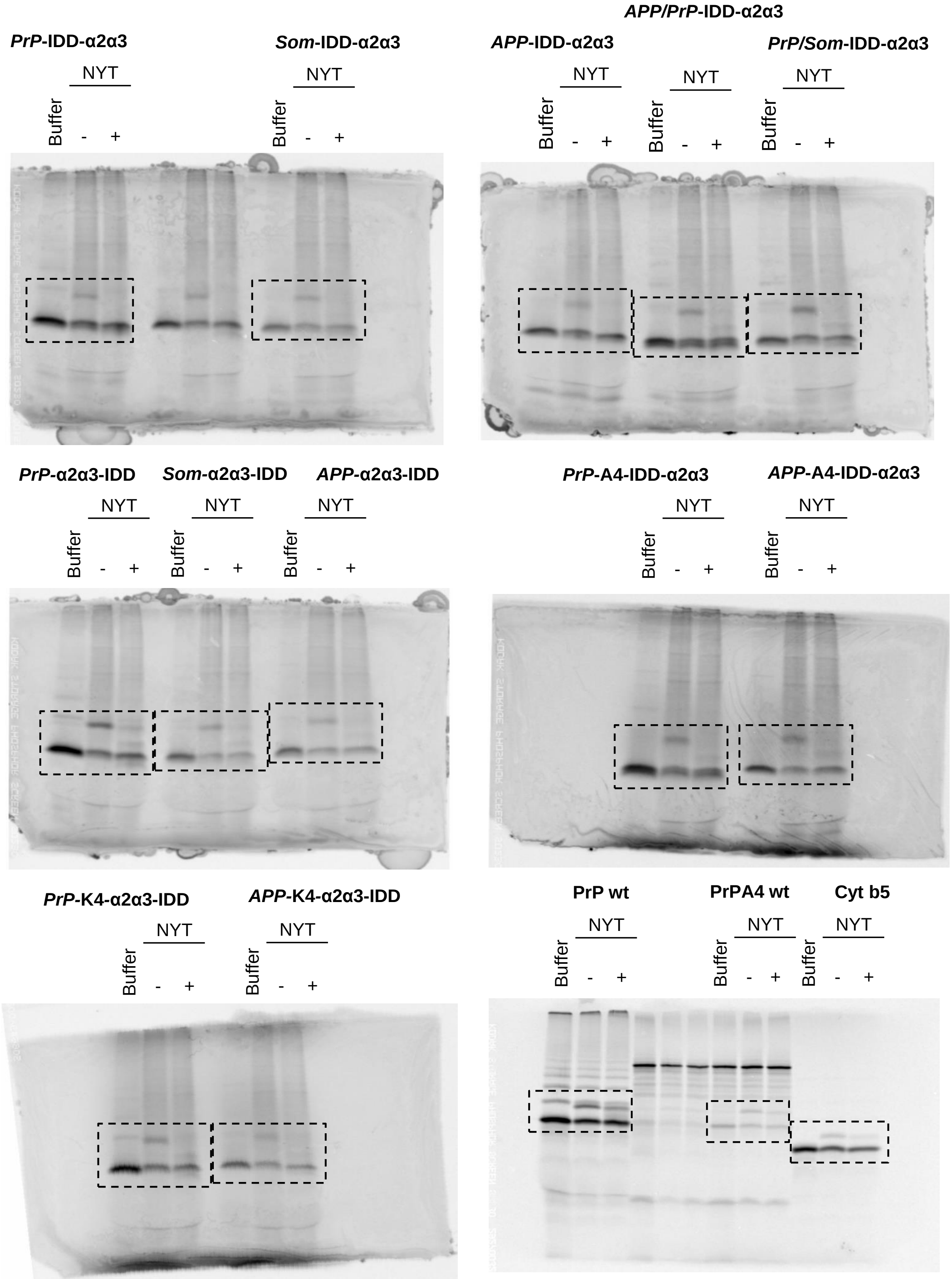

Fig. S4: Complete phosphorimages of cropped gels in Fig. 1. See also Fig. S5.

Biology Open • Supplementary information

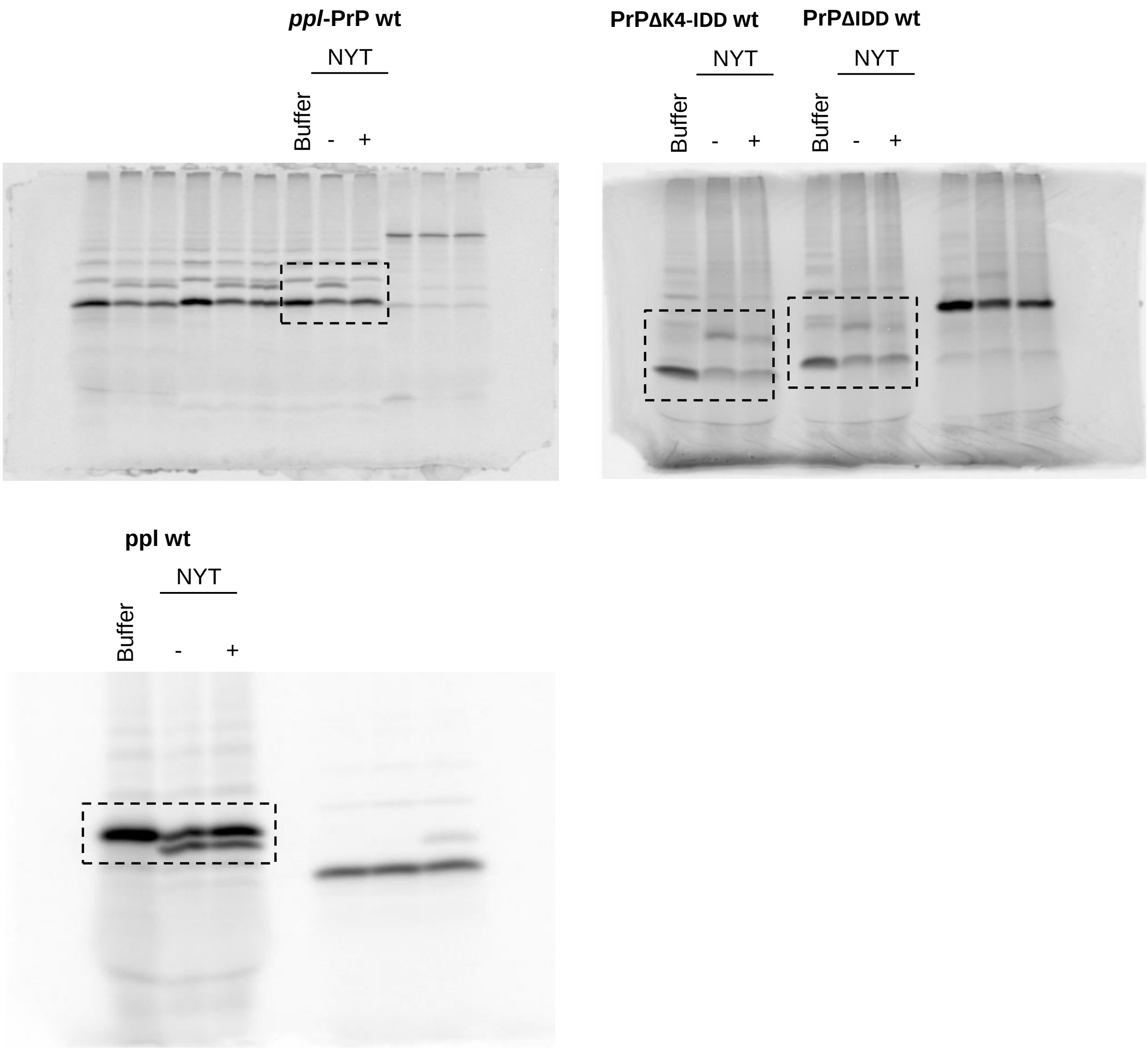

Fig. S5: Complete phosphorimages of cropped gels in Fig. 1. See also Fig. S4.

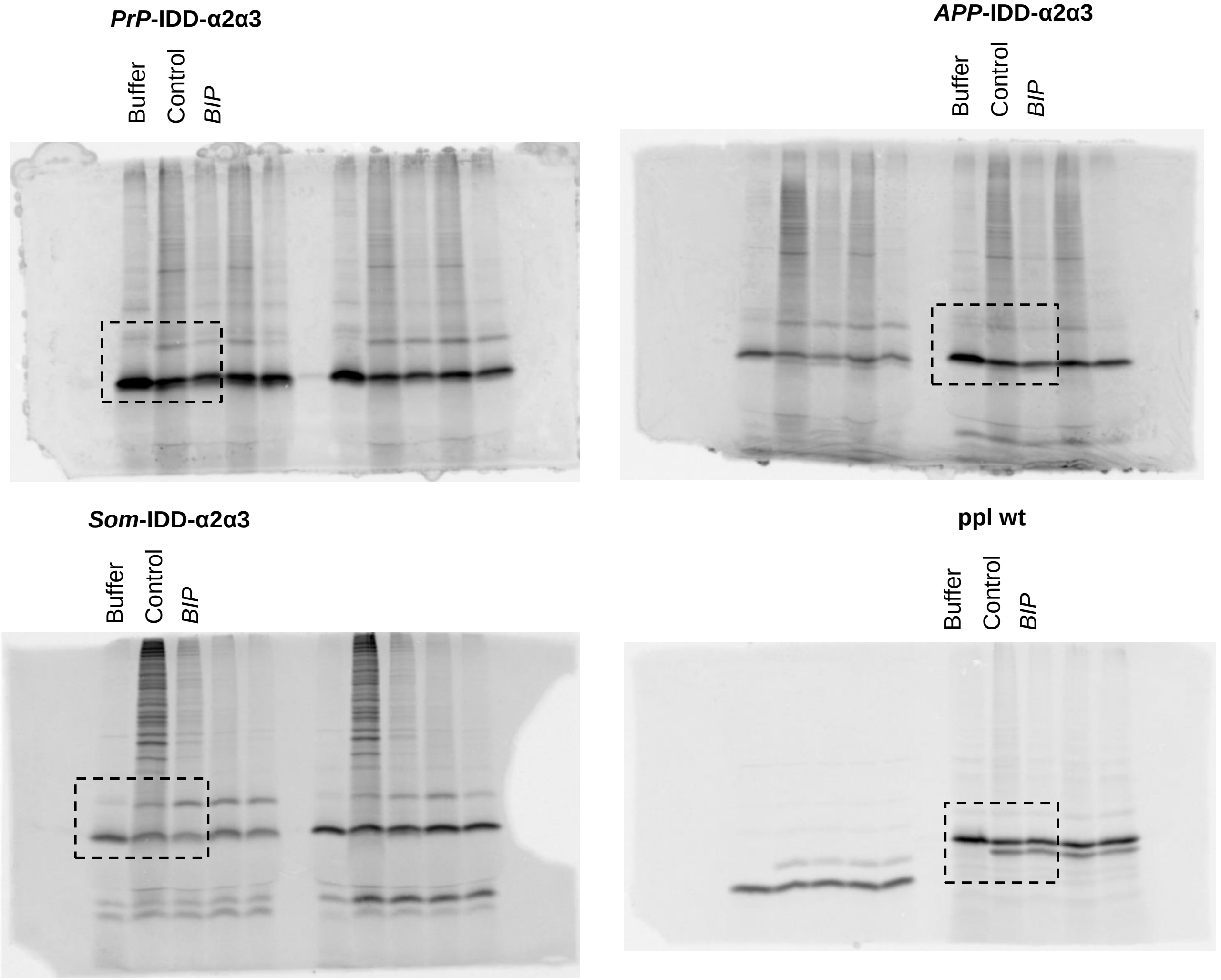

Fig. S6: Complete phosphorimages of cropped gels in Fig. S2A and quantitative data in Fig. 2A.

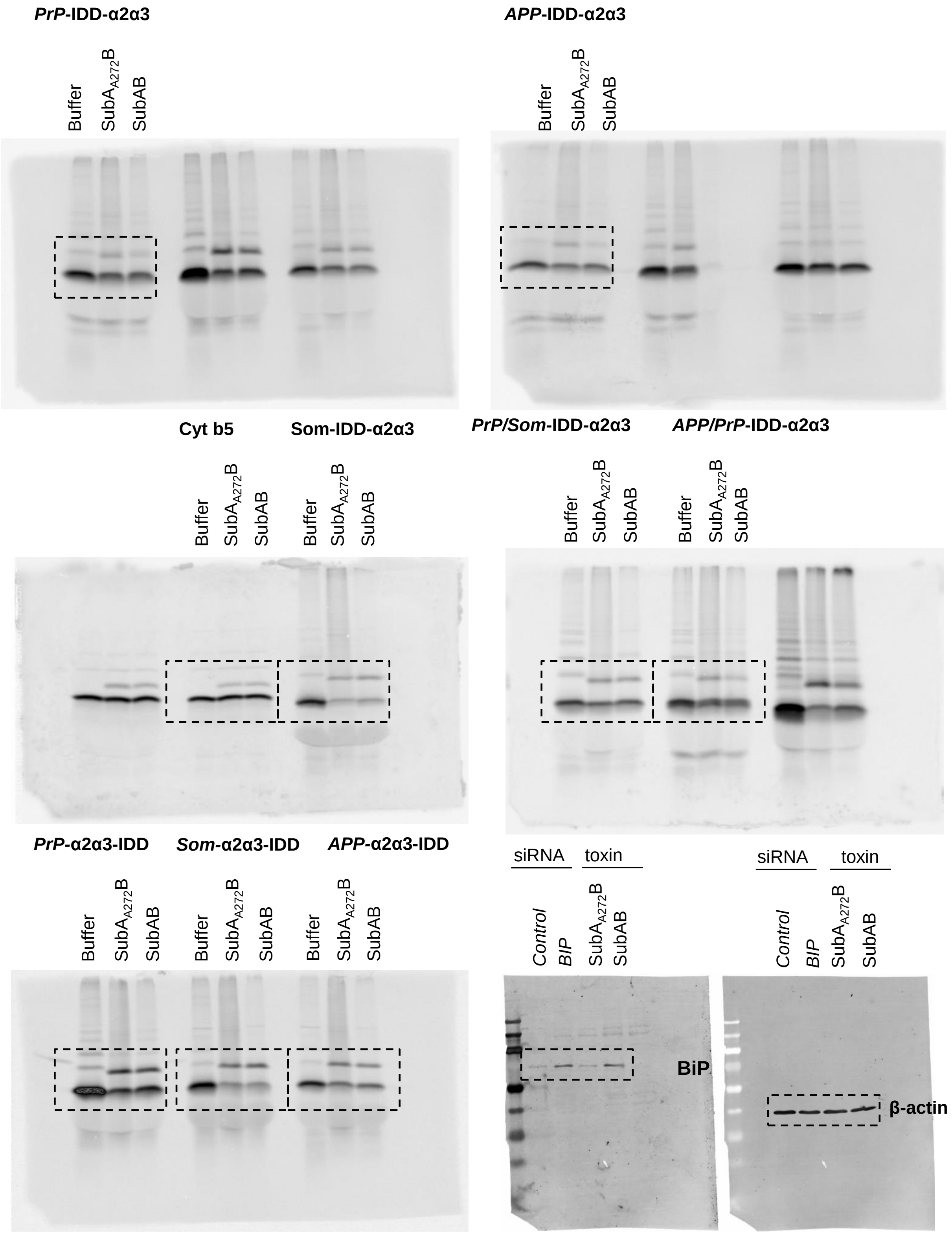

Fig. S7: Representative phosphorimages of cropped gels and Western Blot in Fig. S2B, D-F and quantitative data in Fig. 2. See also Fig. S8.

Biology Open • Supplementary information

Biology Open (2019): doi:10.1242/bio.040691: Supplementary information

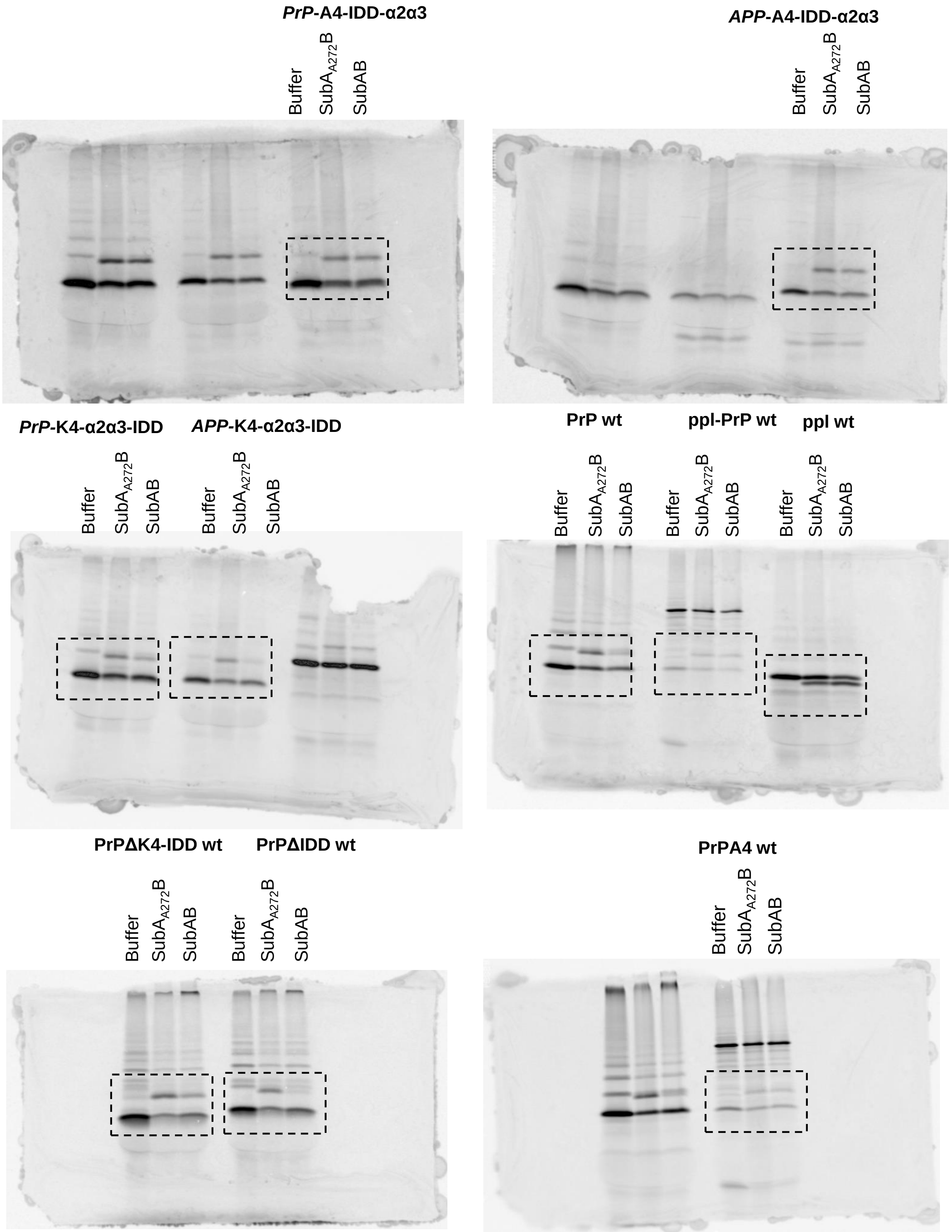

Fig. S8: Representative phosphorimages of cropped gels in Fig. S2E-F and quantitative data in Fig. 2. See also Fig. S7.

Biology Open • Supplementary information

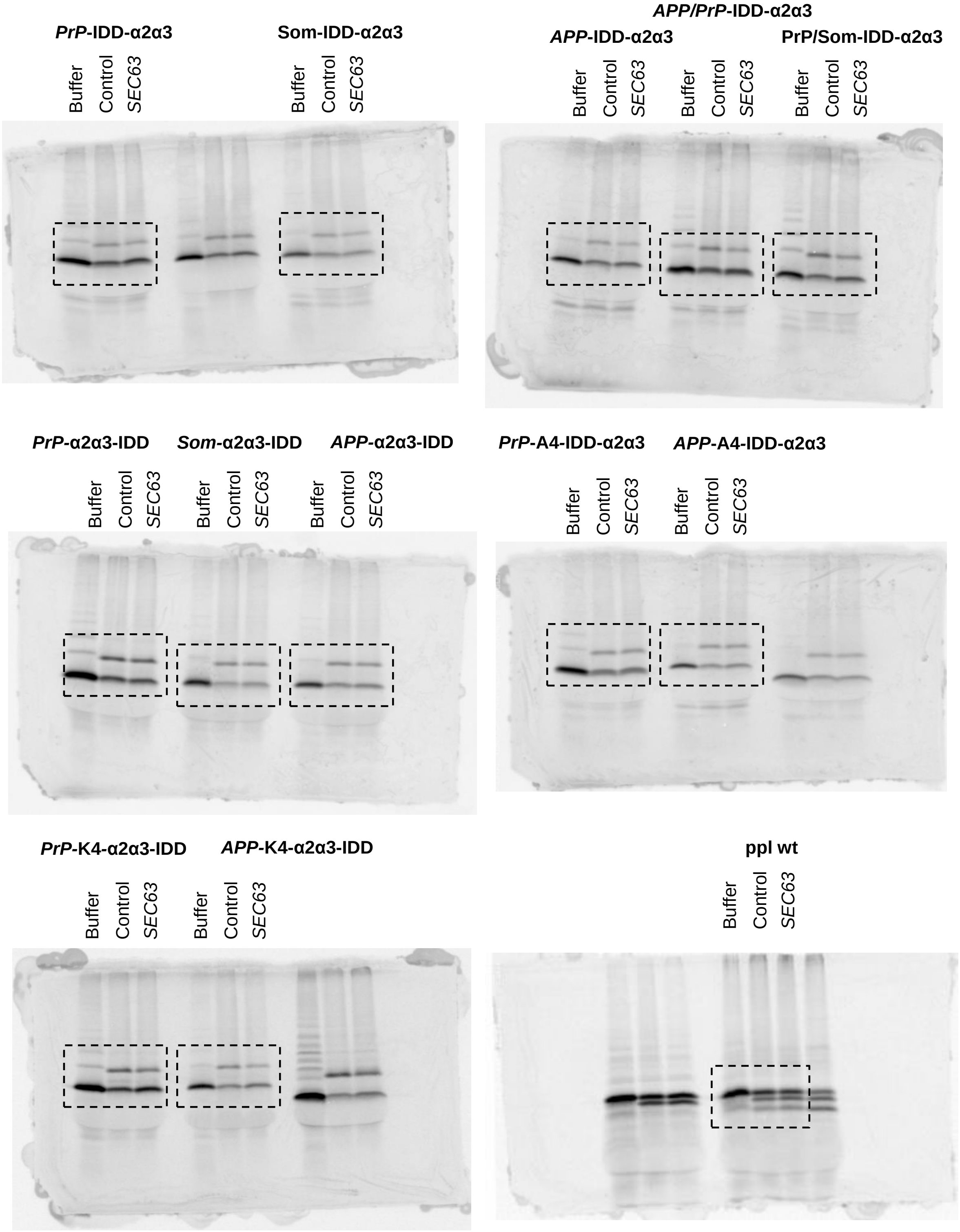

Fig. S9: Representative phosphorimages of cropped gels in Fig. S2G-H and quantitative data in Fig. 2. See also Fig. S10.

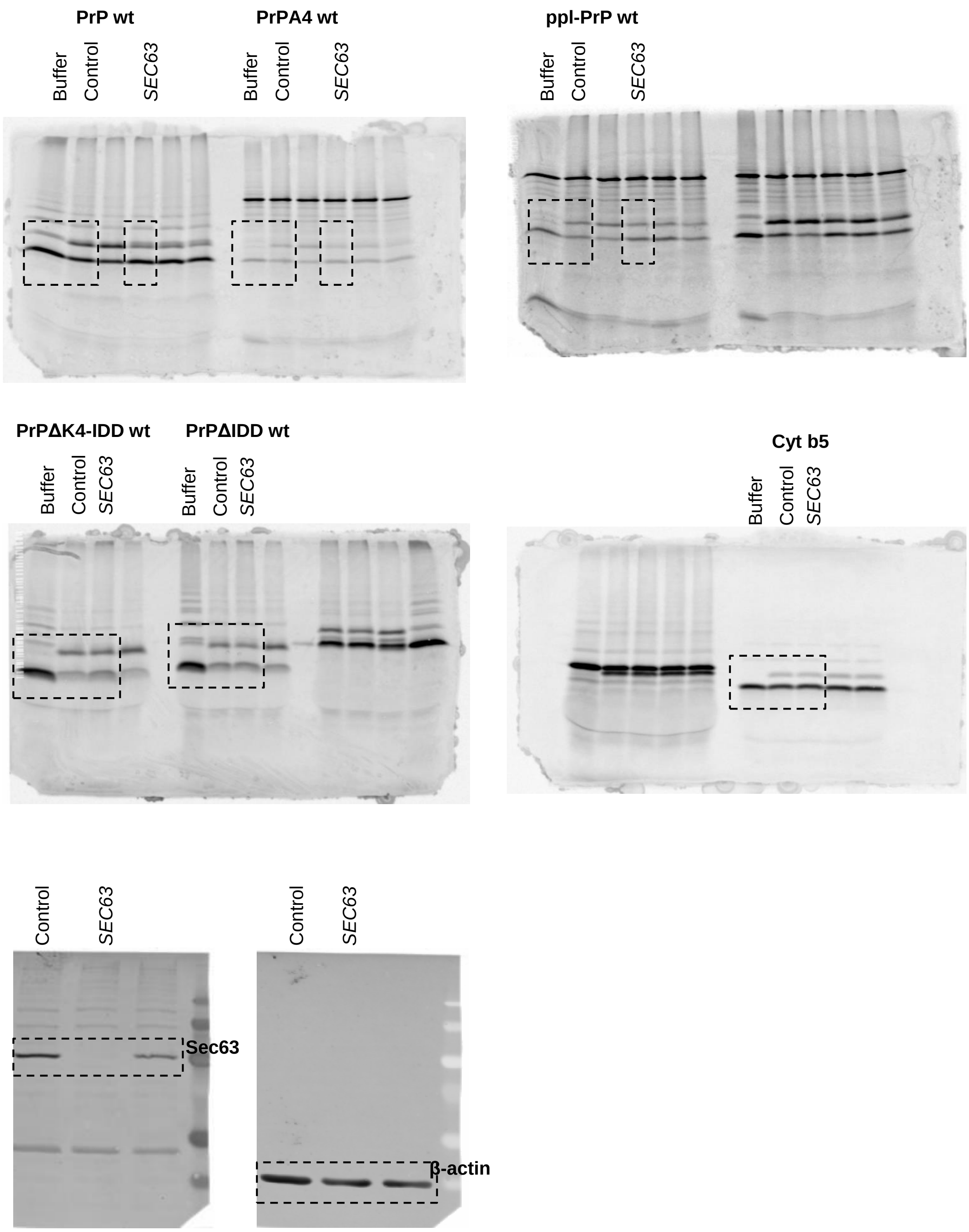

Fig. S10: Representative phosphorimages of cropped gels and Western Blot in Fig. S2C, G-H and quantitative data in Fig. 2. See also Fig. S9.

Biology Open • Supplementary information

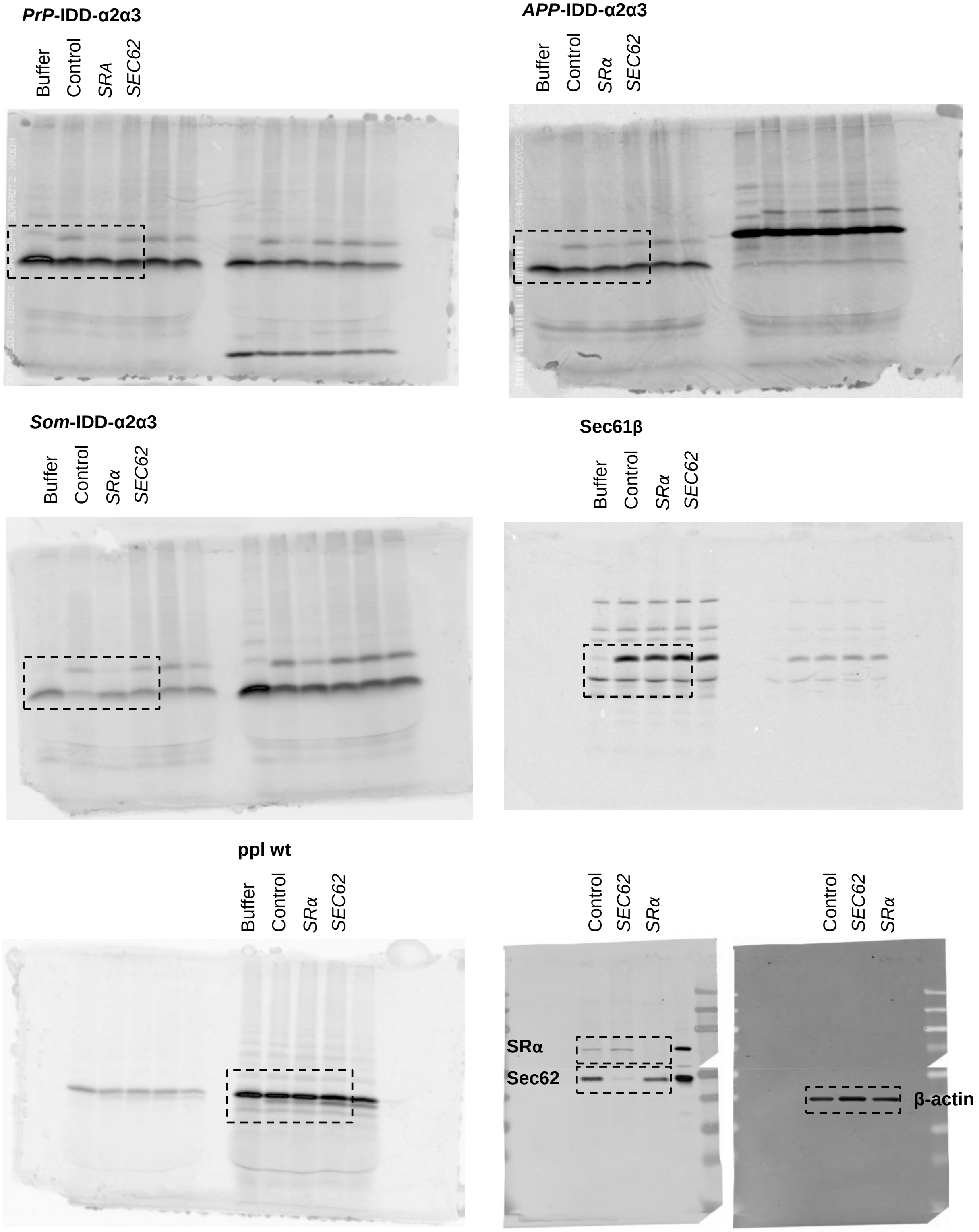

Fig. S11: Representative phosphorimages of cropped gels and Western Blots in Fig. S3A,C and quantitative data in Fig. 3.

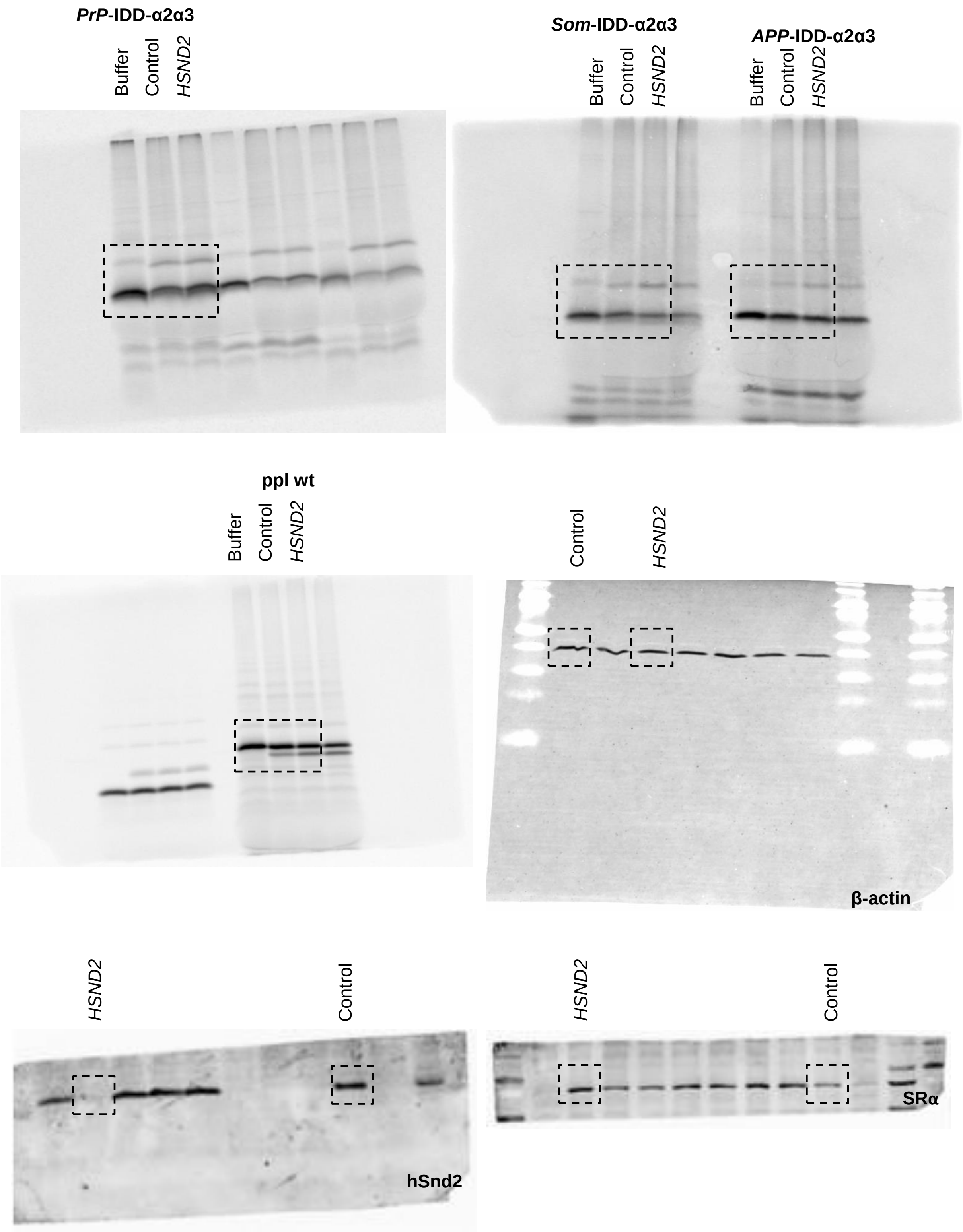

Fig. S12: Representative phosphorimages of cropped gels and Western Blots in Fig. S3B,D and quantitative data in Fig. 3.

Biology Open • Supplementary information

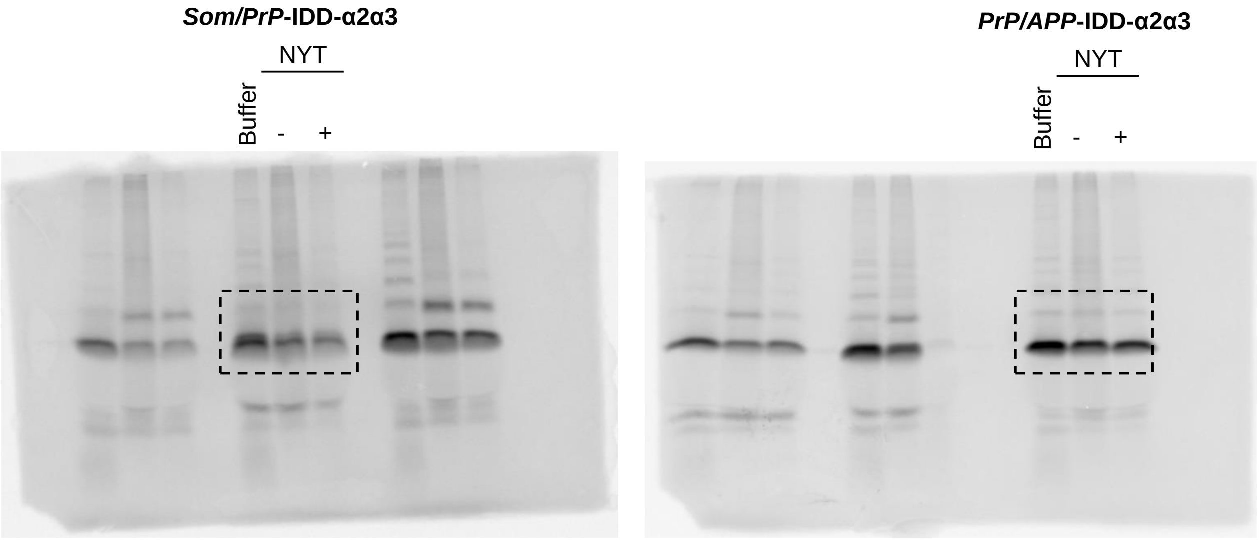

Fig. S13: Complete phosphorimages of cropped gels in Fig. S1.

Biology Open • Supplementary information
